# Development a new thermodynamic model for aqueous polyelectrolyte solutions: extended UNIQUAC coupled with Manning’s theory

**DOI:** 10.3389/fchem.2026.1853816

**Published:** 2026-07-08

**Authors:** Chenxu Duan, Hazem Saeed Zubeiba, Farag Altalbawy, J. Deepak, Anupama Routray, A. Karthikeyan, Harjot Singh Gill, Yashwant Singh Bisht, Rafid Kamal Jameel, Ahmed Aldulaimi, Rafid Jihad Albadr, Mariem Alwan

**Affiliations:** 1 School of Intelligent Manufacturing, Sichuan University Jinjiang College, Meishan, Sichuan, China; 2 The Islamic University of Al Diwaniyah, Al Diwaniyah, Iraq; 3 Department of Chemistry, University College of Duba, University of Tabuk, Tabuk, Saudi Arabia; 4 Department of Mechanical Engineering, School of Engineering and Technology, JAIN (Deemed to be University), Bangalore, Karnataka, India; 5 Department of Mechanical Engineering, Siksha 'O' Anusandhan (Deemed to be University), Bhubaneswar, Odisha, India; 6 Department of Mechanical Engineering, Sathyabama Institute of Science and Technology, Chennai, Tamil Nadu, India; 7 Department of Mechanical Engineering, Chandigarh University, Mohali, Punjab, India; 8 Department of Mechanical Engineering, Uttaranchal Institute of Technology, Uttaranchal University, Dehradun, Uttarakhand, India; 9 College of Dentistry, Alnoor University, Mosul, Iraq; 10 Department of Pharmacy, Al-Zahrawi University, Karbala, Iraq; 11 Ahl Al Bayt University, Kerbala, Iraq; 12 Pharmacy College, Al-Farahidi University, Baghdad, Iraq

**Keywords:** electrolyte, extended UNIQUAC, osmotic coefficient, polyelectrolyte, polyion

## Abstract

A comprehensive thermodynamic framework has been formulated to describe the behavior of aqueous polyelectrolyte solutions using the modified extended UNIQUAC approach. In the proposed model the short-range and long-range interactions have been modeled by the UNIQUAC and Debye-Hückel model, respectively. The effect of counterion condensation was accounted to estimate the effective charge density along the polyion backbone. Counterion condensation was incorporated to determine the reduced, effective linear charge density of the polyelectrolyte backbone. In addition, long-range Coulombic interactions were represented within the extended UNIQUAC model by introducing a Debye-Hückel term, which accounts for both polyion-counterion interactions and the electrostatic contributions arising from interactions among unbound counterions and added salt species. The developed thermodynamic framework was applied to calculate the osmotic coefficient and water activity of a series of aqueous polyelectrolyte solutions. These systems included sodium poly (acrylate) with degrees of polymerization of 5 and 15, ammonium poly (acrylate) with chain lengths of 5, 10, and 20, sodium poly (methacrylate) with polymerization degrees of 6 and 15, and sodium poly (2-acrylamido-2-methyl-1-propanesulfonate) with chain lengths of 2 and 10. The results demonstrate the model’s capability to capture the thermodynamic behavior across different counterions, polymer backbones, and molecular weights. The model formulation explicitly incorporates temperature, polyelectrolyte molecular weight, linear charge density of the polyion, and electrolyte concentration as model variables. The model results indicate that the new approach provides reliable estimates of counterion activity coefficients in systems containing polyelectrolytes. For the seven systems studied, the average relative deviation was 4.59%, showing that the model gives accurate results. Because of its flexible structure, the model can be readily applied to new polyelectrolyte solutions that contain added salts in future work.

## 1 Introduction

Polyelectrolyte solutions have a wide range of applications due to their unique properties. Polyelectrolytes are used as flocculants and coagulants to remove suspended particles and impurities from water (Chen et al., 2024). They help in the clarification of drinking water and wastewater treatment. They enhance paper strength, improve retention of fillers and fibers, and control the drainage rate during papermaking (Hunkeler and Wandrey, 2001). Polyelectrolytes act as thickeners, stabilizers, and film-forming agents in shampoos, conditioners, and other personal care products (Hunkeler and Wandrey, 2001). Polyelectrolyte complexes and nanoparticles are used for controlled drug release (Delgado et al., 2024). As well, they can be used as carriers for DNA and RNA (Delgado et al., 2024). Polyelectrolytes are injected into oil reservoirs to increase the viscosity of the injection water and improve oil displacement (Akyol et al., 2014). The thermodynamic properties of polyelectrolytes are crucial for understanding and predicting their behavior in various applications. Polyelectrolytes often form complexes with oppositely charged polymers, surfactants, or metal ions. The thermodynamics of these complexation processes (such as enthalpy and entropy changes) determine the stability and stoichiometry of the resulting complexes, which is essential in applications like drug delivery, gene therapy, and water treatment (Delgado et al., 2024). Understanding the thermodynamics of polyelectrolyte adsorption is essential for applications such as coatings, adhesives, and surface modification.

Accurate thermodynamic data are essential for developing and validating computational models and simulations of polyelectrolyte systems. These models can be used to predict the behavior of polyelectrolytes under different conditions and to optimize their performance in various applications. Thermodynamic modeling of polyelectrolyte solutions is complex due to the long-range electrostatic interactions between charged monomers and the surrounding counterions. Several approaches have been developed to address these challenges, each with its own strengths and limitations. The Poisson-Boltzmann (PB) theory (Lamm, 2003), Molecular Dynamics (MD) simulations, Monte Carlo (MC) simulations, Density Functional Theory (DFT), Liquid State theories, Equation of State (EoS), and activity coefficient-based models can be utilized to estimate the thermodynamic properties and phase equilibrium calculations of polyelectrolyte containing systems. The Poisson–Boltzmann theory treats ions as point charges in a continuous dielectric and combines the Poisson equation with the Boltzmann distribution. It qualitatively describes polyelectrolyte behavior at low concentrations and charge densities, though the mean-field approximation has limitations. MD simulations explicitly model all atoms and ions in the system and solve Newton’s equations of motion to track their positions and velocities over time. MC simulations use random sampling techniques to explore the configuration space of the system and calculate thermodynamic properties. The MC approach can be used to study phase transitions and other equilibrium properties. DFT is a quantum mechanical method that calculates the electronic structure of the system based on the electron density. It can be used to investigate interactions between polyelectrolytes and ions based on the electronic structure of the system, providing molecular-level insight into their behavior.

Pessoa and Maurer’s extended the Pitzer model likely involved developing a more sophisticated activity coefficient model for polyelectrolytes by incorporating parameters reflecting the polymeric nature of the solute, the complex charge distribution, and the phenomenon of counterion condensation (de Alcântara Pessôa Filho and Maurer, 2008). The resulting model would probably include new adjustable parameters obtained through fitting experimental data. The model would require new interaction parameters specific to the polyelectrolyte and the solvent. These parameters likely would depend on the type of polyelectrolyte, the counterion, and the solvent. The determination of these parameters could have involved fitting the model to experimental data (such as osmotic pressure measurements).

The Yethiraj and Shew model is a theoretical and computational approach primarily used to study the behavior of polyelectrolytes in solution. It utilizes a combination of theoretical concepts and simulation techniques, especially MD simulations (Yethiraj and Shew, 1996). Yethiraj and Shew employ a coarse-grained model in which groups of atoms, such as polymer monomers or solvent molecules, are represented by single beads. Polyelectrolyte chains and explicit counterions are modeled as charged beads, capturing electrostatic interactions, counterion condensation, and screening effects. Electrostatics are typically treated using the Debye-Hückel or Poisson-Boltzmann approach. This framework, combined with molecular dynamics simulations, allows the study of larger systems over longer time scales and provides insights into chain conformation, effective charge, and the influence of ionic strength, key factors for applications like drug delivery, water treatment, and materials science.

S. Naeem and G. Sadowski developed a new equation of state (EoS) based on the SAFT theory for polyelectrolyte containing systems (Naeem and Sadowski, 2010; Naeem and Sadowski, 2011). The pePC-SAFT (polyelectrolyte PC-SAFT) was applied to the phase equilibria of aqueous solutions of poly (sodium acrylate) and poly (ammonium acrylate) with and without added NaCl. Their proposed model was able to predict the vapor–liquid equilibria of polyelectrolyte systems. Recently, the pcPC-SAFT EoS is utilized to describe counterion condensation, complex formation, and liquid-liquid phase separation of polyelectrolyte containing systems (Ascani et al., 2025). Y. Yu et al. extended the electrolyte Non-random two-liquid model (NRTL) to polyelectrolyte containing systems (Yu et al., 2019). The short-range interactions between polyion–ion, ion–ion, ion–water, and polyion–water were described using the local composition framework of the electrolyte NRTL (eNRTL) theory. Y. Yu et al. correlated the counterion activity coefficient and osmotic coefficient for systems containing NaPVS, NaPSS, sodium alginate, and potassium alginate, both with and without added salts. Their results demonstrated that the proposed polyelectrolyte NRTL model can satisfactorily predict the activity coefficients and osmotic coefficients of aqueous polyelectrolyte solutions. S. Ghosh presented a theoretical framework to study interactions between flexible polyelectrolyte chains, showing that bound ions and the entropy of free ions drive complex coacervation (Ghosh, 2025). Their analysis reveals that local factors, such as dielectric constant, strongly influence global thermodynamic behavior and the entropy gain of the system. C. G Lopez et al. focused on the influence of electrostatics on conformational and hydrodynamic properties of polyelectrolyte chains (Carlos et al., 2024). They investigated some key research challenges in order to fully understand the conformation and dynamics of polyelectrolytes in solutions.

These studies demonstrate that electrostatic interactions play a dominant role in determining the thermodynamic behavior of polyelectrolyte solutions. One of the most important electrostatic phenomena is counterion condensation, whereby a fraction of the counterions becomes associated with the charged polymer backbone, reducing its effective charge density. To account for this effect, Manning developed a theory based on the counterion condensation concept for aqueous polyelectrolyte solutions (Manning, 1969a; Manning, 1969b; Manning, 1984). Manning developed a theory to describe the behavior of highly charged polyelectrolytes in solution by accounting for the reduction of the effective polyion charge due to counterion condensation. The theory provides a simple framework for estimating the effective charge density of polyelectrolytes and has been widely used to interpret the thermodynamic behavior of polyelectrolyte solutions. This theory describes how counterions condense onto a polyelectrolyte once its linear charge density exceeds a critical value, partially neutralizing its charge. Driven by electrostatics and entropy, this framework helps explain the behavior of highly charged polyelectrolytes despite its limitations. It provides a simple framework for explaining how counterion condensation influences the effective charge density of the polyion, as well as thermodynamic properties such as osmotic coefficients and counterion activity coefficients in polyelectrolyte solutions. However, it is crucial to be aware of its limitations and consider more advanced approaches for a more complete description of polyelectrolyte behavior in specific systems. Manning’s theory relies on several simplifications, such as assuming a uniformly charged, infinitely long, cylindrical polyion and neglecting the specific interactions between counterions and solvent molecules. As well, real polyions have finite lengths, which can deviate from the predictions of Manning’s theory, especially at the ends of the chain. The theory doesn’t explicitly account for specific ion effects (differences in interactions between different counterions and the polyion). Manning theory assumes uniform charge distribution along the polyion. Real polyions may have variations in charge density.

Nordmeier’s counterion association model (Nordmeier, 1994; Nordmeier, 1995; Nordmeier and Dauwe, 1991) offers a more sophisticated and thermodynamically consistent approach to understanding polyelectrolyte solutions compared to the simpler Manning condensation theory. They investigated the interactions between poly (styrene sulfonate) and counterions in mixed electrolyte systems using the dialysis equilibrium technique (Nordmeier and Dauwe, 1991). The results indicate that the two-variable Manning theory effectively predicts the decrease in divalent counterion condensation as ionic strength increases. It was observed that lead ion binding is strongest in the presence of hydrogen ions and weakest with sodium ions, with the binding of one ion releasing approximately two monovalent ions. While the degree of condensation showed no dependence on polyion concentration, it decreased slightly with rising temperature. Nordmeier provided a comprehensive review of the current state of polyelectrolyte research, focusing on three primary areas: counterion binding phenomena, dynamic processes, and the helix-coil transition of DNA (Nordmeier, 1995). The author critically evaluates leading theoretical models by comparing their predictions with experimental data across a wide range of parameters, including salt concentration, temperature, and solvent composition. A key objective of the work is to identify which phenomena are well-understood theoretically and which remain unresolved, while also aiming to bridge the gap between the study of biological materials like DNA and the physics of synthetic polyelectrolytes to foster closer collaboration between these fields. As well, Nordmeier studied the influence of charge density on polyelectrolyte behavior using poly (acrylic acid) and its copolymers, employing techniques such as activity measurements and Donnan dialysis (Nordmeier, 1994). The results indicate that the experimental data for monovalent and divalent counterions generally agree with Manning’s theory when the charge density parameter is greater than the critical value of 1. However, noticeable deviations appear once this parameter falls below the threshold. These differences are attributed to changes in the flexibility and folding of the polyion, which effectively modify its charge density. In contrast, Nordmeier’s model describes counterion binding to polyelectrolytes as a chemical equilibrium involving specific binding sites and accounts for thermodynamic factors such as enthalpy and entropy. While this approach provides a more realistic description than Manning’s theory, it is also more complex, as it requires additional parameters and an accurate determination of binding constants.

Although several models have been developed to describe polyelectrolyte solutions, each approach has its own limitations. Manning theory successfully describes the counterion condensation phenomenon and the resulting reduction in the effective charge density of the polyion. However, it does not provide a complete thermodynamic framework for representing all interactions present in solution and may not accurately describe concentration-dependent thermodynamic properties. On the other hand, activity-coefficient models such as extended UNIQUAC and NRTL are well suited for describing short-range interactions and solution non-ideality, but they do not explicitly account for counterion condensation. Therefore, there is a need for a thermodynamic model that combines the strengths of both approaches. In this work, Manning’s theory is coupled with the extended UNIQUAC model to account for both counterion condensation and solution non-ideality, providing a more comprehensive description of aqueous polyelectrolyte solutions with and without added salts.

Therefore, the novelty of this work lies in the development of a new thermodynamic framework that combines Manning’s theory with the extended UNIQUAC model, specifically designed for polyelectrolyte systems. This integration allows the extended UNIQUAC model to explicitly account for the counterion condensation phenomenon that is intrinsic to polyelectrolytes. The proposed model was applied to estimate the osmotic coefficient and water activity of ten polyelectrolyte solutions containing NaPA5, NaPA15, NH_4_PA5, NH_4_PA10, NH_4_PA20, NaPMA6, NaPMA15, NaPES2, and NaPES10. Subsequently, the model’s performance was evaluated in the presence of added salts for seven systems: NaPVS–NaCl, NaPSS–NaCl, NaDNA–NaCl, MgPVS–MgCl_2_, MgDNA–MgCl_2_, Na-Alginate–NaCl, and K-Alginate–KCl.

## 2 Theory

### 2.1 The extended UNIQUAC model

The Extended UNIQUAC (Universal Quasi-Chemical) model is a thermodynamic model used to predict activity coefficients and phase equilibria in liquid mixtures, including those containing electrolytes and polymers. It is an extension of the original UNIQUAC model, designed to overcome some of its limitations, particularly when dealing with complex mixtures and systems with significant non-idealities (Thomsen, 1997a). The UNIQUAC accounts for the size and shape differences between molecules through a combinatorial term. This term is based on the assumption that molecules are arranged on a quasi-lattice. It uses parameters like the van der Waals volumes and surface areas of the components to estimate the entropy of mixing. The residual term accounts for the energetic interactions between molecules. It uses adjustable parameters (interaction parameters) to represent the interactions between different component pairs in the mixture. The Extended UNIQUAC model incorporates several enhancements to address the limitations of the original UNIQUAC model. The extended model explicitly accounts for electrostatic interactions between ions in electrolyte solutions. This is typically done by incorporating terms based on the Debye-Hückel theory or other suitable approaches for modeling long-range electrostatic interactions. As well, the model may include ion-specific parameters to capture the different interactions of various ions with the solvent and other components in the mixture. The extended UNIQUAC can be applied to a wide range of mixtures, including electrolyte solutions, polymer solutions, and mixtures of organic compounds. This model can provide reasonable predictions of activity coefficients and phase equilibria, even for systems with limited experimental data. Accurate parameter estimation is crucial for obtaining reliable predictions. This can be challenging, especially for complex mixtures with many components.

The extended UNIQUAC model (Equations 1–8) is expressed as follows (Thomsen, 1997a):

$$G^{E}=G_{Combinatorial}^{E}+G_{Residual}^{E}+G_{Debye-Huckel}^{E}$$ (1)

$$\frac{G_{Residual}^{E}}{RT}=-\sum_{i=1}^{N}x_{i}q_{i}\ln\left(\sum_{k=1}^{N}\theta_{k}\psi_{ki}\right)$$ (2)

$$\frac{G_{Combinatorial}^{E}}{RT}=\sum_{i=1}^{N}x_{i}\ln\left(\frac{\phi_{i}}{x_{i}}\right)-\frac{z}{2}\sum_{i=1}^{N}q_{i}x_{i}\ln\left(\frac{\theta_{i}}{\phi_{i}}\right)$$ (3)

$$\frac{G_{Debye-Huckel}^{E}}{RT}=-4x_{w}M_{W}A\left[\ln\left(1+bI^{0.5}\right)-bI^{0.5}+0.5Ib^{0.5}\right]/b^{3}$$ (4)

$$\psi_{ki}=\exp\left(-\frac{\left(u_{ki}-u_{ii}\right)}{T}\right)$$ (5)

$$u_{ki}=u_{ki}^{0}+u_{ki}^{t}\left(T-298.15\right)$$ (6)

$$\phi_{i}=\frac{x_{i}r_{i}}{\sum_{j}x_{j}r_{j}}$$ (7)

$$\theta_{i}=\frac{x_{i}q_{i}}{\sum_{j}x_{j}q_{j}}$$ (8)

In Equation 4, *G* ^*E*^ is the molar excess Gibbs energy, *M*_*w*_ is the molar mass of water (kg/mol), b = 1.5 (kg/mol)^1/2^ is a constant. A is the Debye–Hückel parameter. The Equation 9 gives the temperature dependence of A at temperatures up to 500 K (Sander et al., 1986):

$$A=[1.3131+1.335\times 10^{-3}\left(T\left(K\right)-273.15\right)+1.164\times 10^{-5}\;({\left.T\left(K\right)-273.15)\right]}^{2}{\left(kg/mol\right)}^{0.5}$$ (9)

In the upper equations, *G* ^*E*^ is Gibbs free energy, *z* is coordination number with a value of 10; *x*_*i*_ is mole fraction; ϕ_i_ is volume fraction and θ_i_ is the surface area fraction. As well, *r*_i_ and *q*_*i*_ are the volume and surface area parameters. *u*_*ii*_ is the interaction energy between similar components in an equilibrium system and *u*_*ij*_ is the interaction energy between different components. In Equation 4 *I* is the ionic strength:

$$I=0.5\sum_{i=1}^{ions}m_{i}z_{i}^{2}$$ (10)

In Equation 10, *z*_*i*_ is the charge and *m*_*i*_ is the molality of ion *i.*

In the next section the Manning theory has been described.

### 2.2 Manning theory

Manning’s theory provides a valuable framework for understanding polyelectrolyte behavior. In Manning theory several core concepts must be defined.

- Polyion: A highly charged polymer chain, such as a DNA molecule or a synthetic polyelectrolyte.
- Counterions: Ions of opposite charge to the polyion, present in the solution to maintain electroneutrality.
- Condensation: A fraction of the counterions associate closely with the polyion due to strong electrostatic attraction, effectively neutralizing a portion of the polyion’s charge. This is not a chemical binding; the counterions are still mobile, though localized near the polyion.
- Effective charge: The remaining, uncondensed counterions contribute to the effective charge of the polyion, which is significantly lower than the bare charge of the polyion.

The central prediction of Manning’s theory is that condensation occurs if a dimensionless parameter, *ξ (x*_*i*_ *)*, exceeds a critical value:

$$\xi =\frac{e^{2}}{\varepsilon k_{B}Tb}$$ (11)

In Equation 11 *e* is the elementary charge, *ε* is the permittivity of the solvent, *k*_*B*_ refers to the Boltzmann constant, and *b* is the average distance between charged monomers along the polyion chain. If *ξ* > 1, condensation occurs. This means that if the electrostatic interactions between the charged monomers are strong enough relative to the thermal energy (*k*_*B*_ *T*), counterion condensation will take place.

The condensed counterions effectively screen the polyion’s charge, leading to a reduction in its effective charge and, consequently, the electrostatic interactions between polyions. Manning condensation has significant consequences for the physical properties of polyelectrolyte solutions. The reduced electrostatic interactions between polyions can lead to lower solution viscosity. The screening of charge can affect the solubility and aggregation behavior of polyelectrolytes. The osmotic pressure is affected because the effective concentration of charged species is lower due to condensation.

The electrostatic potential between a counterion *i* and a polyion *p* is given by Equation 12 (Yu et al., 2019):

$$u_{ip}=-\frac{2z_{i}e\beta }{\varepsilon }\ln\frac{\rho }{\rho_{0}}$$ (12)

where *z*_*i*_ and ε are the charge number of point charge *I* and the dielectric constant of the solvent, respectively. $\rho_{0}$ refers to the reference distance where the potential is zero (Manning, 1969b). β is charge density (Equations 13, 14):

$$\beta =\frac{z_{p}e}{b}$$ (13)

$$b=\frac{L}{P}$$ (14)

*L* refer to the total length of the polyion, and *P* is the number of charged segments in the polyion. *z*_*p*_ is the charge number of charged segment on polyion *p* and *b* is the average distance between the closest charged segments of the polyion (Yu et al., 2019).

Using the aforementioned electrostatic potential, the total Helmholtz excess free energy for aqueous polyelectrolyte solutions was derived using Equations 15–17 (Yu et al., 2019):

$$\frac{A^{E}}{k_{B}VT}=-\frac{1}{2}\xi c_{p}\ln\left[\lambda \left(c_{p}+2c_{coion}\right)\right]\;for\;\xi <1$$ (15)

$$\frac{A^{E}}{k_{B}VT}=-\frac{1}{2}\frac{c_{p}}{\xi }\ln\left[\lambda \left(\frac{c_{p}}{\xi }+2c_{coion}\right)\right]\;for\;\xi >1$$ (16)

$$\lambda =\frac{4\pi e^{2}}{\varepsilon k_{B}T}$$ (17)

where A^E^, c_p_, c_coion_, and V refer to total Helmholtz excess free energy, number concentration of polyelectrolyte, number concentration of coion, and volume of solution, respectively.

Y. Yu et al. derived the molality-based excess Gibbs free energy of Manning theory as follows (Yu et al., 2019):

$$\frac{G^{E}}{MwRT}=-\frac{1}{2}\xi m_{p}\ln\left[\lambda^{\prime }\left(m_{p}+2m_{coion}\right)\right]\;for\;\xi <1$$ (18)

$$\frac{G^{E}}{MwRT}=-\frac{1}{2}\frac{m_{p}}{\xi }\ln\left[\lambda^{\prime }\left(\frac{m_{p}}{\xi }+2m_{coion}\right)\right]\;for\;\xi >1$$ (19)

λ′=4πF2d s1000εRT (20)

In Equations 18–20 F refer to Faraday’s constant, *d*_*s*_ is the solvent density and *m*_*i*_ is the molality of species *i*.

The mobile ion activity coefficient and osmotic coefficient in Manning theory can be derived as follows (Yu et al., 2019):

$$\ln\left(\gamma_{i}^{*M}\right)=-\frac{\xi X}{2\left(X+2\right)}\;for\;\xi <1$$ (21)

$$\phi^{M}=1-\frac{\xi X}{2\left(X+2\right)}\;for\;\xi <1$$ (22)

In the case of condensation of polyelectrolytes ( ξ>1 ), the following equations were obtained (Yu et al., 2019):

$$\ln\left(\gamma_{counterion}^{\text{*}\text{M}}\right)=\ln\left(\frac{\frac{X}{\xi }+1}{X+1}\right)-\frac{\frac{X}{\xi }}{2\left(\frac{X}{\xi }+2\right)}\;for\;\xi >1$$ (23)

$$\ln\left(\gamma_{coion}^{\text{*}\text{M}}\right)=-\frac{\frac{X}{\xi }}{2\left(\frac{X}{\xi }+2\right)}\;for\;\xi >1$$ (24)

$$\phi^{M}=\frac{\frac{\xi X}{2}+2}{X+2}\;for\;\xi >1$$ (25)

In the aforementioned equations, X refers to the ratio of the number of moles of polyion

Charged segments, and the number of moles of added salt (X = n_p_/n_salt_).

All above equations were developed in the presence of added salts. When there is no added salt, the Manning’s limiting law yields constant values for osmotic coefficient and activity coefficient given by Equations 26–29 as follows:

$$\ln\left(\gamma_{counterion}^{\text{*}\text{M}}\right)=-\frac{\xi }{2}\;for\;\xi <1\text{ and }X\to \infty$$ (26)

$$\ln\left(\gamma_{counterion}^{\text{*}\text{M}}\right)=-\frac{1}{2}-\ln\left(\xi \right)\;for\;\xi >1\text{ and }X\to \infty$$ (27)

$$\phi^{M}=1-\frac{\xi }{2}\;for\;\xi <1\text{ and }X\to \infty$$ (28)

$$\phi^{M}=\frac{1}{2\xi }\;for\;\xi >1\text{ and }X\to \infty$$ (29)

Manning’s limiting law is a fundamental result in polyelectrolyte theory, providing a quantitative prediction for the osmotic coefficient at high charge densities, highlighting the dominant role of electrostatic interactions and counterion condensation in these systems. However, it is essential to be aware of its limitations and consider more advanced models for a more complete description of polyelectrolyte behavior in specific systems. In polyelectrolyte solutions, the osmotic pressure is primarily determined by the concentration of free ions (both polyions and counterions). Due to Manning condensation, a significant fraction of counterions is condensed onto the polyion, reducing the effective charge of the polyion and the number of free ions in solution. The osmotic coefficient is inversely proportional to the Manning parameter ξ. As ξ increases (higher charge density), the osmotic coefficient decreases, indicating a larger degree of counterion condensation. The higher the charge density, the more counterions condense, reducing the number of osmotically active particles in the solution. The limiting osmotic coefficient, according to Manning’s law, is independent of the polymer concentration. This prediction is valid in the regime where the electrostatic interactions are so strong that they dominate the osmotic behavior.

In the next section the new model based on the extended UNIQUAC model coupled with the Manning theory has been described.

### 2.3 New model for polyelectrolyte solutions (PE-Extended UNIQUAC)

In section 2.2, the Manning limiting law has been described. Manning’s limiting law is a valuable theoretical framework, while often shows discrepancies when compared to experimental observations. These deviations arise because of the various idealizations and simplifications made in the theory. Manning’s limiting law predicts that the osmotic coefficient is independent of polymer concentration at sufficiently high charge densities. Experimental data often show a dependence on polymer concentration, especially at higher concentrations. The theory neglects polymer-polymer interactions. At higher concentrations, these interactions become significant. As well, even with counterion condensation, the remaining effective charge on the polyions can lead to significant repulsions. This theory treats all counterions of the same valence as identical, without considering their specific chemical nature, while different counterions (even with the same valence) can have different affinities for the polyion, leading to varying degrees of binding or association. Because the theory ignores the detailed interactions between counterions and the polyion. It must be noted that, smaller ions might be able to get closer to the polyion, and more polarizable ions may interact more strongly. On the other hand, the theory assumes an infinitely long polyion, while real polymers have a finite length. The ends of the polyion have a different electrostatic environment than the middle. Counterion condensation near the ends can be different. The number of monomers is also very important for the shorter polymers where the ends can have a greater effect on the overall charge of the polyelectrolyte. This can have many effects (such as end effects and flexibility).

These discrepancies mean that, the quantitative predictions of Manning’s limiting law are often inaccurate, especially in complex systems. The theory provides a qualitative understanding of the role of charge density and counterion condensation but should not be relied upon for precise quantitative predictions.

In this work, a new thermodynamic model is developed to account for both long-range and short-range interactions in polyelectrolyte solutions. Long-range interactions between ions and between ions and polyions are described using the Debye–Hückel theory. Short-range interactions among all components are modeled through the residual and combinatorial contributions of the extended UNIQUAC model. The phenomenon of counterion condensation is incorporated via Manning’s theory. By differentiating the excess Gibbs free energy with respect to the mole fraction of each species, the activity coefficients are derived accordingly (Thomsen, 1997b; Thomsen, 2005):

$$\ln\left(\gamma_{i}^{*}\right)=\ln\left(\gamma_{i}^{DH}\right)+\ln\left(\gamma_{i}^{Residual}\right)-\ln\left(\gamma_{i}^{Residual,\infty }\right)+\ln\left(\gamma_{i}^{Combinetorial}\right)-\ln\left(\gamma_{i}^{Combinetorial,\infty }\right)+\ln\left(\gamma_{i}^{Manning}\right)$$ (30)

The activity coefficient of Manning contribution was defined in Equations 21–27. The activity coefficient of Debye-Huckel has been obtained as follows:

$$\ln\left(\gamma_{i}^{DH}\right)=-\frac{z_{i}^{2}AI^{0.5}}{\left(1+bI^{0.5}\right)}$$ (31)

The corresponding term for water is:

$$\ln\left(\gamma_{w}^{DH}\right)=2AMw\left[1+bI^{0.5}-{\left(1+bI^{0.5}\right)}^{-1}-2\ln\left(1+bI^{0.5}\right)\right]/b^{3}$$ (32)

where M_w_ is the water molecular weight based on the kg/mol. The combinatorial contribution to the activity coefficient of component *i* is (Thomsen, 1997b; Thomsen, 2005):

$$\ln\left(\gamma_{i}^{Combinetorial}\right)=\ln\left(\frac{\phi_{i}}{x_{i}}\right)+1-\frac{\phi_{i}}{x_{i}}-5q_{i}\left[\ln\left(\frac{\phi_{i}}{\theta_{i}}\right)+1-\frac{\phi_{i}}{\theta_{i}}\right]$$ (33)

$$\ln\left(\gamma_{i}^{Residual}\right)=q_{i}\left[1-\ln\left(\sum_{l=1}^{N}\theta_{l}\psi_{li}\right)-\sum_{j=1}^{N}\left(\theta_{j}\psi_{ij}/\sum_{l=1}^{N}\theta_{l}\psi_{lj}\right)\right]$$ (34)

The infinite dilution terms are obtained by setting *x*_*w*_ *= 1* in the above equations:

$$\ln\left(\gamma_{i}^{Combinetorial,\infty }\right)=\ln\left(\frac{r_{i}}{r_{w}}\right)+1-\frac{r_{i}}{r_{w}}-5q_{i}\left[\ln\left(\frac{r_{i}q_{w}}{r_{w}q_{i}}\right)+1-\frac{r_{i}q_{w}}{r_{w}q_{i}}\right]$$ (35)

$$\ln\left(\gamma_{i}^{Residual,\infty }\right)=q_{i}\left[1-\ln\left(\psi_{wi}\right)-\psi_{wi}\right]$$ (36)

The activity coefficient for water is calculated in the extended UNIQUAC model by summation of the three terms as follows (Thomsen, 1997b; Thomsen, 2005):

$$\ln\left(\gamma_{w}\right)=\ln\left(\gamma_{w}^{DH}\right)+\ln\left(\gamma_{w}^{Residual}\right)+\ln\left(\gamma_{w}^{Combinetorial}\right)+\ln\left(\gamma_{w}^{Manning}\right)$$ (37)

Therefore, Equations 30–37 can be used to calculate the counterion activity coefficient, water activity, and osmotic coefficient of aqueous polyelectrolyte solutions.

## 3 Results and discussion

### 3.1 Model parameters

The Extended UNIQUAC model is a powerful tool for modeling the thermodynamic properties of complex mixtures. Its flexibility comes from the introduction of temperature-dependent interaction parameters and the ability to incorporate specific interactions. However, the success of the model depends on the availability of reliable experimental data and careful parameter estimation. In the extended UINIQUAC model *rᵢ* (van der Waals volume parameter) represents the relative van der Waals volume of molecule *i*. It is usually calculated from group contribution methods or taken from experimental data. It reflects the size of the molecule and its contribution to the combinatorial entropy. *q*_*i*_ (van der Waals surface area parameter) represents the relative van der Waals surface area of molecule *i*. It is similarly calculated from group contribution methods or experimental data. It reflects the number of intermolecular contact sites. The binary interaction parameter reflects the energetic interactions between species in solution. It accounts for deviations from ideal mixing arising from specific interactions among the components that cannot be fully described by the molecular volume and surface area parameters alone.

**FIGURE 1:**
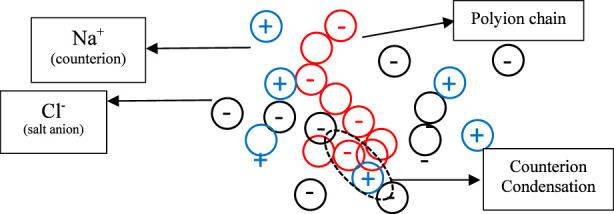
Schematic of NaPVA-NaCl-H_2_O polyelectrolyte solution with added salts (NaCl).

In a polyelectrolyte solution, assuming that counterion condensation has occurred, the system can be considered to consist of counterions, anions, polyions, condensed neutral segments, and water. In Figure 1, NaPVA-NaCl-H_2_O system as an example has been depicted.

As shown in Figure 1, five species can be considered. Take the NaPVA-NaBr-H_2_O system as an example. H_2_O (1), polyion PVA^−^ (2), counterion NA^+^ (3), anion Cl^−^ (4), and condensed neutral segment NaPVA (5) are available in systems.

The Manning parameter (*ξ*) in polyelectrolyte theory quantifies the linear charge density of the polyelectrolyte chain relative to the Bjerrum length in the solvent. It essentially tells you how strong the electrostatic interactions are in the system. This is the average charge per unit length along the polymer chain. For a fully charged polyelectrolyte, it depends on the spacing between charged monomers along the chain. Adjusting the Manning parameter is crucial. The Manning parameter strongly influences the polymer’s conformation. Accurate knowledge or control of the Manning parameter is essential for reliable theoretical modeling and computer simulations of polyelectrolyte systems.

In this work, the Manning parameter, the volume parameter (*rᵢ*), surface area parameter (*q*_*i*_) of polyion and condensed neutral segment, and the binary interaction coefficient between polyion-water, counterion-condensed neutral segment, and water-condensed neutral segment have been adjusted using the following objective function (OF):

$$OF=w_{1}\sum_{i}{\left[\frac{\gamma_{i}^{+,\exp}-\gamma_{i}^{+,calc}}{\gamma_{i}^{+,\exp}}\right]}^{2}+w_{2}\sum_{i}{\left[\frac{\phi^{\exp}-\phi^{calc}}{\phi^{\exp}}\right]}^{2}$$ (38)

where $w_{1}$ and $w_{2}$ refer to weight parameters of OF; refer to Table 2. *exp* and *calc* refer to experimental and calculated values. Volume parameter (*rᵢ*) and surface area parameter (*q*_*i*_) of ions and polyion, condensed neutral segment, and water have been reported in Table 1. The *rᵢ* and *q*_*i*_ of polyion and condensed neutral segment have been adjusted using the osmotic coefficient or counterion activity coefficient experimental data. In the case of ions and water, the volume and surface area parameters have been extracted in the published articles (Thomsen, 1997b; Thomsen, 2005). The binary interaction coefficient (*u*_*ij*_) between all components have been reported in the Supplementary Material.

**TABLE 1:** Volume parameter (*rᵢ*) and surface area parameter (*q*_*i*_) of PE-Extended UNIQUAC.

Components	*rᵢ*	*q*_*i*_	Components	*rᵢ*	*q*_*i*_
H_2_O	0.92	1.40	NaPA5	3.85	30.21
${Na}^{+}$	1.4034	1.199	NaPA15	5.37	52.84
$K^{+}$	1.86	1.71	NaPES2	2.94	19.40
${Mg}^{2+}$	3.55	0.805	NaPES10	3.61	35.54
${NH}_{4}^{+}$	4.815	4.6028	NaPMA6	5.37	52.84
${Cl}^{-}$	10.82	10.4	NaPMA15	6.03	59.33
${PA5}^{-}$	3.015	29.85	NH_4_PA5	7.47	87.89
${PA15}^{-}$	4.313	41.53	NaDNA	4.17	141.80
${PES2}^{-}$	2.645	21.41	NaPVS	2.77	87.48
${PES10}^{-}$	3.154	37.57	MgPVS	2.50	5.01
${PMA6}^{-}$	4.312	41.53	NaPSS	8.88	189.43
${PMA15}^{-}$	5.048	63.29	MgDNA	1.12	6.338
${DNA}^{-}$	2.929	84.65	SANa	6.58	92.30
${PVS}^{-}$	3.793	14.88	PAK	5.77	121.60
${PSS}^{-}$	7.927	135.65	​	​	​
${SA}^{-}$	7.820	10.09	​	​	​
${KA}^{-}$	6.986	58.30	​	​	​

In Table 2, the Manning parameters of each aqueous polyelectrolyte solutions have been reported.

**FIGURE 2:**
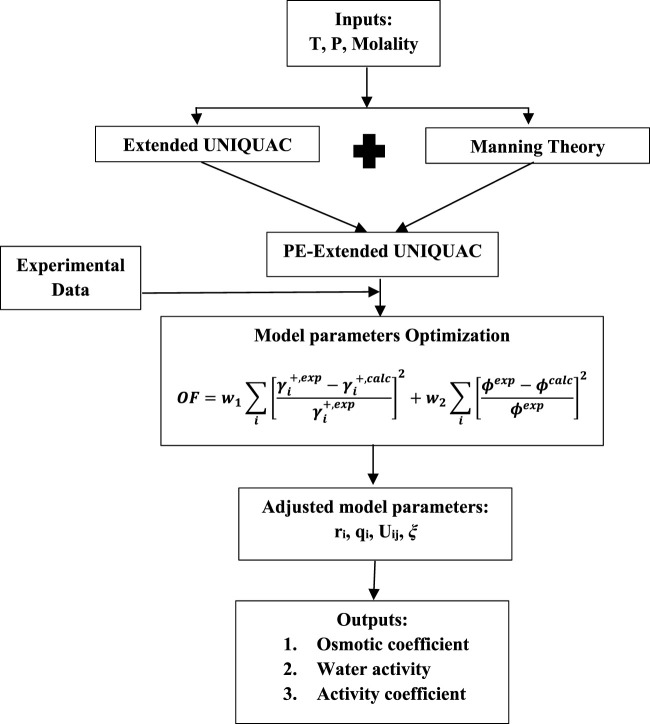
Flowchart for calculations and optimization methods of PE-Extended UNIQUAC.

In Figure 2, a flowchart for calculations and optimization methods of the proposed model has been depicted.

As shown in Table 2, the effect of added salts on seven polyelectrolyte containing systems have been studied. The results show that, the regressed Manning parameter for NaPA15 (ξ = 5.054) is higher than that for NaPA5 (ξ = 4.164), although both polymers are composed of identical acrylate repeat units. According to classical Manning theory, the charge density parameter is determined by the spacing between charged groups along the polymer backbone and therefore would be expected to remain constant for polymers having the same chemical structure. However, in the present work, ξ is treated as an effective parameter obtained through regression of thermodynamic data within the extended UNIQUAC framework. The increase in ξ with increasing degree of polymerization suggests that the regressed parameter captures not only the intrinsic linear charge density but also additional chain-length-dependent effects that are not explicitly represented in the model. These effects may include finite-chain behavior, changes in chain conformation, variations in counterion distribution around the polyion, and differences in intermolecular interactions as the molecular weight increases. Consequently, the regressed Manning parameter should be interpreted as an effective charge-density parameter that reflects the combined influence of electrostatic and thermodynamic contributions required to reproduce the experimental data. A similar trend can be observed for NaPES, where ξ increases from 3.141 for NaPES2 to 3.962 for NaPES10. The relatively modest increase in ξ compared with the much larger increase in degree of polymerization indicates that no simple linear relationship exists between ξ and chain length. Instead, the results suggest that ξ approaches a limiting value as the polymer chain becomes longer. Additional experimental data covering a wider molecular-weight range would be required to establish a quantitative correlation between the effective Manning parameter and the degree of polymerization.

**TABLE 2:** Regressed Manning parameter and literature data of aqueous polyelectrolyte solutions at room temperature for parameters optimization.

Components	Salt	*ξ*	w_1_	w_2_	Exp. Data
NaPA5	N/A	4.164	0	1	Ise and Okubo (1965), Lammertz et al. (2008)
NaPA15	N/A	5.054	0	1	Ise and Okubo (1965), Lammertz et al. (2008)
NaPES2	N/A	3.141	0	1	Lammertz et al. (2008)
NaPES10	N/A	3.962	0	1	
NaPMA6	N/A	5.054	0	1	
NaPMA15	N/A	5.318	0	1	
NH_4_PA5	N/A	4.316	0	1	​
NaDNA	NaCl	1.876	1	0	Lyons and Kotin (1965)
NaPVS	NaCl	2.231	1	0	
MgPVS	MgCl_2_	9.87	1	0	
NaPSS	NaCl	3.068	1	0	
MgDNA	MgCl_2_	3.681	1	0	
SA	NaCl	1.123	1	0	Podlas and Ander (1970)
PA	KCl	1.072	1	0	

In the next sections the model performance in two cases (with and without added salt) has been described.

### 3.2 Aqueous polyelectrolyte solutions without added salt

Without the screening effect of added salt, electrostatic interactions between charged monomers on the polyelectrolyte chain and between different chains become very long-range. This makes it difficult to truncate these interactions in modeling. As well, the distribution of counterions around the polyelectrolyte is highly non-uniform and crucial to the solution behavior. Accurate modeling of this distribution is essential. The chain conformation (how the polyelectrolyte chain folds and extends) is strongly influenced by electrostatic repulsions, leading to more extended conformations compared to neutral polymers or polyelectrolytes in high salt concentrations. In this work seven free-salt polyelectrolyte solutions containing NaPA5, NaPA15, NaPES2, NaPES10, NaPMA6, NaPMA15, and NH4PA5 have been studied. The activity of water (or osmotic coefficient) is one of the most important properties of polyelectrolyte solutions. The isopiestic method was utilized for the activity of water in aqueous solutions of the aforementioned synthetic polyelectrolytes. The isopiestic method is a comparative method for determining the water activity of a solution by equilibrating it with a reference solution of known water activity in a closed system. The principle is that at equilibrium, the vapor pressure of water (and thus the water activity) is the same in both solutions. The isopiestic method directly measures the water activity, which is a fundamental thermodynamic property of the solution. When performed carefully, the isopiestic method can provide highly accurate water activity data. Unlike some other methods, the isopiestic method doesn’t require assumptions about the behavior of the solute (the polyelectrolyte) in the solution. This is particularly important for polyelectrolytes, which can exhibit complex behavior due to their charge and polymeric nature.

In Table 3, the average relative deviation (ARD%) of new model (PE-Extended UNIQUAC) has been reported.

**TABLE 3:** The average relative deviation (ARD%) of osmotic coefficient and water activity of PE-Extended UNIQUAC.

Components	PE-extended UNIQUAC	
​	ϕ	$a_{w}$
NaPA5	5.6	0.21
NaPA15	5.8	0.23
NaPES2	7.5	0.36
NaPES10	8.2	0.19
NaPMA6	9.2	0.43
NaPMA15	6.9	0.18
NH_4_PA5	5.1	0.17
Average Error	6.8	0.25

As shown in Table 3, the P- Extended UNIQUAC model can estimate the osmotic coefficient and water activity of seven polyelectrolyte solutions in the absence of added salt. In Table 2, the *w*_*2*_ parameters in OF has been set to 1 for the aforementioned seven polyelectrolyte systems. Therefore, the model parameters have been adjusted using the osmotic coefficient experimental data. The adjusted model parameters have been utilized to predict the water activity. The results show that, the proposed model can correlate/predict the osmotic coefficient and water activity of aqueous polyelectrolyte solutions without added salt, satisfactory.

In Supplementary Appendices A, B, the fully dissociated approach has been investigated for polyelectrolyte-containing systems to assess model performance and to evaluate the effect of Manning theory. In this context, two models are considered: the Extended UNIQUAC model and the electrolyte PC-SAFT (ePC-SAFT) equation of state (Chapman et al., 1990; Gross and Sadowski, 2001; Held et al., 2008). In the fully dissociated approach, electrolytes in solution are assumed to dissociate completely into their constituent ions, with no formation of ion pairs or associated species. Detailed descriptions of the modeling approach and the corresponding results are provided in Supplementary Appendices A, B.

**FIGURE 3:**
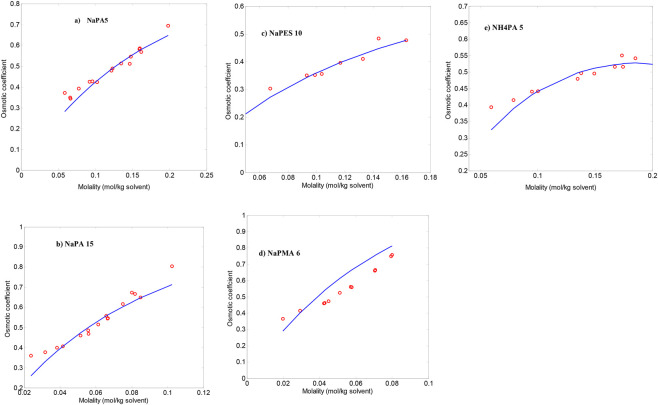
Osmotic coefficient of polyelectrolyte solutions at 298.15 K. Lines are model estimation and symbols refer to experimental data (Table 2 last column). **(a)** NaPA5. **(b)** NaPA 15. **(c)** NaPES 10. **(d)** NaPMA 6. **(e)** NH4PA 5.

In Figure 3, the osmotic coefficient of polyelectrolyte solutions has been depicted and compared to experimental data.

As shown in Figure 3, the PE-Extended UNIQUAC model effectively estimates the osmotic coefficient of polyelectrolyte solutions without added salt. Figures 3a,b present the estimated osmotic coefficients for two sodium polyacrylate (PA) polyelectrolytes: NaPA5 and NaPA15. These compounds are sodium salts of polyacrylic acid and are widely used in various applications due to their unique properties as superabsorbent polymers and polyelectrolytes. Understanding the activity and osmotic coefficients of polysodium acrylate in solution is essential for optimizing its performance in different applications, such as water retention, drug delivery, and industrial processes. As the concentration of polysodium acrylate increases, interactions among the charged polymer chains can lead to increased non-ideality. The presence of other ions can screen electrostatic interactions, thus modifying the activity coefficient. As well, the ionization of the functional groups in polysodium acrylate is pH-dependent, which can modify the polymer’s charge density and its interaction with water. In this work, the PE-Extended UNIQUAC model accurately accounts for both long-range and short-range interactions among charged–charged, charged–uncharged, and uncharged–uncharged species. The charge densities of NaPA5 and NaPA15 were determined to be 4.16 and 5.05, respectively. The average number-average molar masses (Mn) of NaPA5 and NaPA15 are approximately 2600 g/mol and 6900 g/mol, respectively. The results indicate that the proposed model satisfactorily captures the charge density behavior of polyelectrolytes; specifically, an increase in the molecular weight of the polyelectrolyte corresponds to an increase in charge density. As shown in Table 3, the average absolute relative deviations (ARD%) for the osmotic coefficient and water activity of NaPA5 were 5.6% and 0.21%, respectively, while those for NaPA15 were 5.8% and 0.23%. These results demonstrate good agreement with the experimental data.

In Figure 3c, the predicted osmotic coefficient of poly (sodium ethylene sulfonate) (NaPES10) is compared with experimental data. NaPES10 has an average molar mass of approximately 6900 g/mol and consists of 53 monomer units. This water-soluble polyelectrolyte carries a strong negative charge due to the presence of sulfonate groups. NaPES is utilized in various applications where its polyelectrolyte properties (particularly its ability to influence solution behavior) are advantageous. For example, it can inhibit scale formation in industrial water systems by interfering with the crystallization of minerals such as calcium carbonate. Additionally, NaPES is used in drug delivery systems to modify drug release profiles and enhance bioavailability. PES derivatives are also being investigated as electrolyte additives to enhance the performance and safety of lithium-ion batteries. Understanding the interactions between PES and metal ions is essential for applications such as scale inhibition and water treatment. In this context, accurate estimation of the thermodynamic properties of NaPES-containing systems is crucial. As shown in Figure 3c, the proposed model successfully correlates the osmotic coefficient of NaPES10 up to a polyelectrolyte molality of 0.2 mol/kg. The average absolute relative deviations (ARD%) for the osmotic coefficient and water activity were found to be 8.2% and 0.19%, respectively.

In Figure 3d, the osmotic coefficient and water activity of NaPMA6 were estimated using the PE-Extended UNIQUAC model. The average absolute relative deviations (ARD%) for the osmotic coefficient and water activity were 9.2% and 0.43%, respectively. The larger deviation observed for NaPMA6 may be attributed to the short polymer chain length and the presence of the methyl group in the methacrylate repeat unit, which can introduce local steric and conformational effects that are not explicitly represented in the current model formulation. NaPMA6 has an average molar mass of approximately 6100 g/mol and consists of 56 monomer units. This polyelectrolyte is commonly used as a scale inhibitor to prevent the formation of mineral deposits in industrial water systems. Additionally, NaPMA6 and similar polyelectrolytes are employed in enhanced oil recovery (EOR) processes to increase the viscosity of injected water, thereby improving oil displacement in reservoirs. Accurately modeling the osmotic coefficient of NaPMA solutions is particularly challenging due to the need to account for electrostatic interactions, excluded volume effects, and ion correlations. The results of this study demonstrate that the proposed PE-Extended UNIQUAC model is capable of effectively correlating and predicting the osmotic coefficient and water activity of NaPMA6 polyelectrolyte solutions.

**FIGURE 4:**
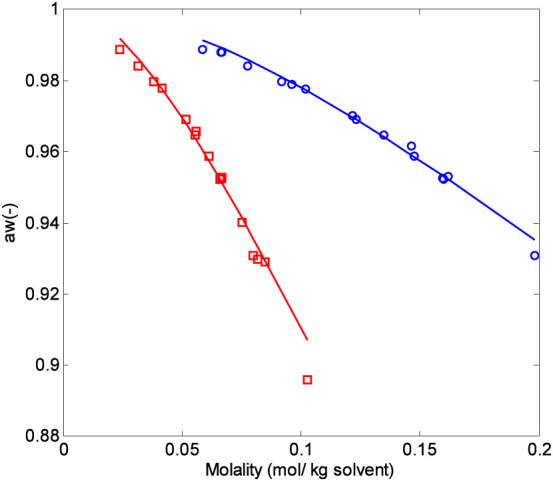
Water activity in NaPA5 (□) and NaPA15 (○) solutions. Lines are PE-Extended UNIQUAC model and symbols refers to experimental data.

The water activity can be predicted using the PE-Extended UNIQUAC model satisfactory. Figure 4 illustrates the water activity profiles of NaPA5 and NaPA15.

As shown in Figure 4, the model can predict the water activity in NaPA5 and NaPA15 solutions, accurately.

**FIGURE 5:**
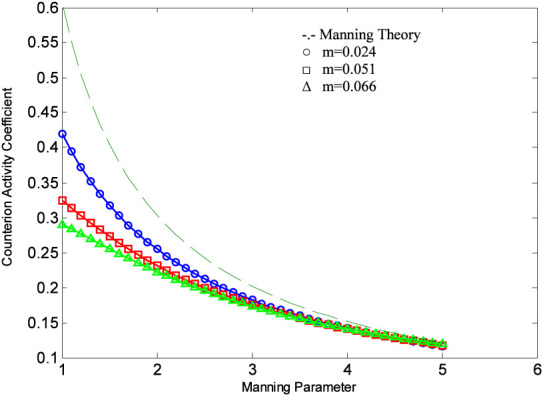
The effect of charge density on counterion activity coefficient in NaPA15 aqueous solutions. M refers to polyelectrolyte molality.

It must be noted that, Manning’s limiting law suggests that the counterion activity coefficients in a polyelectrolyte solution approach a constant value at high ionic strength. This constant value is not simply 1. The specific constant depends on the charge density of the polymer and the type of counterions. Manning’s limiting law suggests the counterion activity coefficients of polyelectrolyte-water binary depend only on Manning parameter ( ξ ). The experimental data indicates that the counterion activity coefficients should also depend on the polyelectrolyte concentrations (Nagasawa and Kagawa, 1957). In Figure 5, the effect of charge density (Manning parameter) on counterion activity coefficient of NaPA15 has been illustrated.

The charge density essentially dictates the strength of the electrostatic field emanating from the polymer chain. This field, in turn, significantly influences the distribution and activity of counterions in the solution. A high charge density on the polyelectrolyte backbone leads to a phenomenon called “counterion condensation” (or Manning condensation). This means that a significant fraction of the counterions is drawn very close to the polymer chain to neutralize the excessive charge. The counterions that are condensed around the polyelectrolyte have a reduced effective concentration in the bulk solution. This is because they are strongly associated with the polymer and less free to participate in other interactions (like contributing to osmotic pressure or conductivity). As a result, the counterion activity coefficient decreases. The activity coefficient reflects the departure from ideal solution behavior. A lower activity coefficient signifies stronger interactions between the ions and the polyelectrolyte. On the other hand, a low charge density means the electrostatic field around the polyelectrolyte is weaker. Therefore, the counterions are more dispersed in the solution and behave more ideally. The counterion activity coefficient is higher. As shown in Figure 5, a higher charge density on the polyelectrolyte leads to stronger electrostatic attraction of counterions, increased counterion condensation, and a lower counterion activity coefficient. The counterions are less free and more strongly associated with the polymer. Conversely, a lower charge density results in weaker attraction, less condensation, and a higher activity coefficient. In Supplementary Appendices A, B comparison between electrolyte PC-SAFT model and Extended UNIQUAC and the PE-Extended UNIQUAC has been studied.

### 3.3 Aqueous polyelectrolyte solutions with added salt

Adding salt to an aqueous polyelectrolyte solution introduces a screening effect that weakens the electrostatic interactions, causes the polyelectrolyte chain to coil, and increases the counterion activity coefficient. This is because the added salt screens the electrostatic interactions between the polyelectrolyte and its counterions, making the counterions behave more ideally. At very high salt concentrations, the electrostatic interactions are so effectively screened that the polyelectrolyte behaves almost as if it were uncharged. At high salt concentrations, the counterion activity coefficient approaches unity; however, this behavior should not be interpreted as thermodynamic ideality, since the reference state is defined at infinite dilution. The magnitude of these effects depends on the salt concentration, the charge density of the polyelectrolyte, and the specific properties of the salt ions. Depending on the specific polyelectrolyte and salt, the addition of salt can either increase or decrease the solubility of the polyelectrolyte. In some cases, the addition of salt can lead to salting out, where the polyelectrolyte precipitates out of the solution. In some cases, the addition of salt can promote the aggregation of polyelectrolyte chains. This is particularly true if the salt ions have a strong affinity for the polyelectrolyte chains. In the absence of salt, the electrostatic repulsions between charged monomers along the polyelectrolyte chain cause it to stretch out into a more extended conformation. As salt is added and the electrostatic repulsions are screened, the polyelectrolyte chain becomes more flexible and tends to coil up into a more compact conformation. It must be noted that, the ions from the added salt compete with the polyelectrolyte’s counterions for association with the charged polymer. At sufficiently high salt concentrations, the added salt can effectively displace the polyelectrolyte’s counterions.

The extent of charge screening is often characterized by the Debye length. The Debye length represents the distance over which electrostatic interactions are significant. As the salt concentration increases, the Debye length decreases, meaning that the electrostatic interactions are screened over shorter distances. Therefore, the presence of added salt significantly alters the electrostatic interactions and consequently influences the properties of the polyelectrolyte solution. In this study, seven polyelectrolyte-containing systems with added salt were examined to evaluate the performance of the proposed model. Specifically, the counterion activity coefficients in the NaDNA–NaCl–H_2_O, NaPVS–NaCl–H_2_O, MgPVS–MgCl_2_–H_2_O, NaPSS–NaCl–H_2_O, MgDNA–MgCl_2_–H_2_O, sodium alginate (SA)–NaCl–H_2_O, and potassium alginate (KA)–KCl–H_2_O systems were modeled using the PE-Extended UNIQUAC framework.

In Table 4, the ARD% values of counterion activity coefficient of the aforementioned systems have been reported.

**TABLE 4:** The average relative deviation (ARD%) of counterion activity coefficient of PE-Extended UNIQUAC.

Components	PE-extended UNIQUAC	Exp. Data
​	$\gamma_{+}$	​
NaDNA-NaCl-H_2_O	5.51	Lyons and Kotin (1965)
NaPVS-NaCl-H_2_O	1.71	
MgPVS-MgCl_2_-H_2_O	1.47	
NaPSS-NaCl-H_2_O	0.16	
MgDNA-MgCl_2_-H_2_O	14.2	
SA-NaCl-H_2_O	2.72	Podlas and Ander (1970)
PA-KCl-H_2_O	6.41	
Average Error	4.59	​

The NaDNA-NaCl-H_2_O system is a very important and well-studied system in biophysics and biochemistry because it mimics the conditions found in biological cells. Understanding the interactions and properties of this system is crucial for understanding DNA’s behavior. Sodium salt of DNA, a highly charged polyelectrolyte. Each phosphate group on the DNA backbone carries a negative charge, which is neutralized by Na^+^ counterions. DNA has a very high charge density due to the closely spaced phosphate groups. The addition of NaCl screens the electrostatic repulsions between the negatively charged phosphate groups on the DNA backbone. This screening effect is more pronounced at higher NaCl concentrations. At low NaCl concentrations, the electrostatic repulsions between the phosphate groups cause the DNA to be in a more extended or unfolded conformation. The high charge density of DNA leads to significant counterion condensation. The fraction of condensed counterions depends on the NaCl concentration. The NaDNA-NaCl-H_2_O system is a simplified model of the cellular environment. The concentration of ions (especially Na^+^, K^+^, and Cl^−^) in cells plays a crucial role in regulating DNA structure, stability, and interactions with other molecules (such as proteins). Understanding the behavior of DNA in the presence of salt is essential for understanding many biological processes.

**FIGURE 6:**
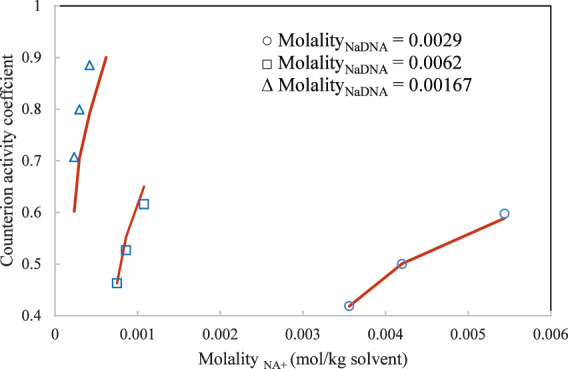
The PE-Extended UNIQUAC results and experimental data (Lyons and Kotin, 1965) of counterion activity coefficient for NaDNA-NaCl-H_2_O ternary solutions.

In Figure 6, counterion activity coefficient in NaDNA-NaCl-H_2_O system has been estimated using the PE-Extended UNIQUAC model.

As shown in Figure 6, the PE-Extended UNIQUAC can estimate the counterion activity coefficient in three polyelectrolyte concentration. The Manning parameters of NaDNA-NaCl-H_2_O ternary solutions has been obtained 1.87. The results show that, the proposed model estimates the counterion activity coefficient over a wide range of salt concentrations. The slight underestimation of the counterion activity coefficient at low Na^+^ molality may be due to the stronger influence of electrostatic interactions in dilute solutions. In this concentration range, the activity coefficient is very sensitive to the effective charge of the polyion and the distribution of counterions around it. Since these effects are approximated using Manning’s theory and the Debye–Hückel model, small deviations between the model and experimental data may occur.

It must be noted that, the x-axis shows the total amount of Na^+^ ion in the solution (sum of polyelectrolyte and salt concentrations). As shown in Equation 23, when the salt concentration increases (lower X-value), the counterion activity coefficient tends to increase.

**FIGURE 7:**
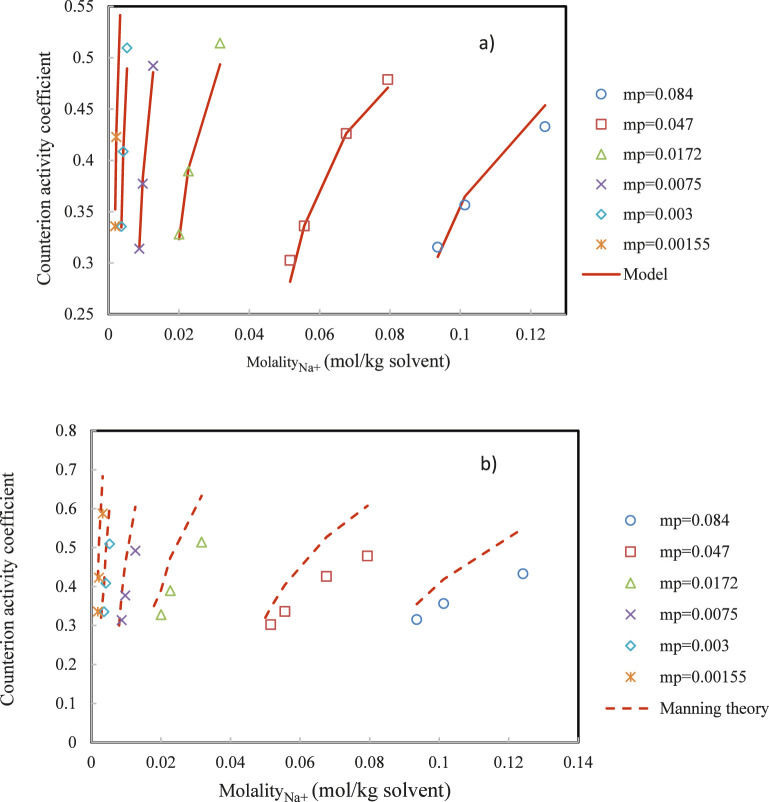
**(a)** The PE-Extended UNIQUAC results and **(b)** Manning theory (ζ = 2.05). Symbols are experimental data (Lyons and Kotin, 1965) of counterion activity coefficient for NaPVS-NaCl-H_2_O ternary solutions. *M*_*p*_ refers to polyelectrolyte molality.

In Figure 7, the counterion activity coefficient of NaPVS-NaCl-H_2_O system has been estimated and compared to experimental data.

The NaPVS-NaCl-H_2_O system refers to an aqueous solution containing sodium poly (vinyl sulfonate) (NaPVS) and sodium chloride (NaCl). Similar to the NaDNA system, this is a polyelectrolyte solution, but with different characteristics and applications. Sodium poly (vinyl sulfonate), a synthetic, highly charged polyelectrolyte. Each vinyl sulfonate unit carries a negative charge neutralized by a Na^+^ counterion. NaPVS is commonly used as a model polyelectrolyte due to its well-defined structure and relatively simple synthesis. It is a strong polyelectrolyte, meaning it is fully charged over a wide pH range. As the NaCl concentration increases, the screening effect causes the NaPVS chain to coil up and become more compact. The polymer transitions to a more globular conformation. This is similar to the behavior of DNA at high salt. The Manning parameters of NaPVS-NaCl-H_2_O system has been adjusted 2.231. The average ARD% of counterion activity coefficient has been obtained 2.46%. As shown in Figure 7a, the PE-Extended UNIQUAC model can estimate the counterion activity coefficient in the presence of added salts accurately. In Figure 7b, the Manning theory has been utilized to predict the counterion activity coefficient with ζ = 2.05. The results show that, the Manning theory cannot estimate the counterion activity coefficient accurately. It should be noted that the Manning parameter (ξ = 2.05) used in Figure 7b was determined using Manning theory alone. In contrast, the ξ values reported in Table 2 were obtained within the coupled PE–Extended UNIQUAC framework, where ξ was evaluated together with the extended UNIQUAC model parameters. Consequently, the ξ value used in Figure 7b and those reported in Table 2 originate from different modeling approaches and are therefore not directly comparable.

As indicated in Equation 23, the counterion activity coefficient of Manning theory is a function of X = n_p_/n_s_ and ζ. It must be noted that, the counterion activity coefficient in polyelectrolyte solutions is depended on the short-range and long-range interactions between charged and uncharged particles. In the new model (PE-Extended UNIQUAC), the short-range interaction between components was considered by combinatorial and residual contribution of UNIQUAC model. On the other hand, the long-range interaction was considered by Debye–Hückel term.

**FIGURE 8:**
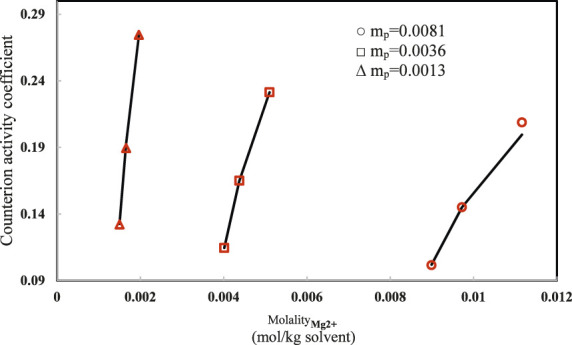
The PE-Extended UNIQUAC results (lines) and experimental data (symbols) (Lyons and Kotin, 1965) of counterion activity coefficient for MgPVS-MgCl_2_-H_2_O ternary solutions.

In Figure 8, counterion activity coefficient in MgPVS-MgCl_2_-H_2_O system has been estimated and compared to experimental data.

The MgPVS-MgCl_2_-H_2_O system is a more complex and interesting system than the NaPVS-NaCl-H_2_O system. The divalent nature of Mg^2+^ leads to a range of unique phenomena that are not observed with monovalent cations. Accurate modeling of this system requires the use of advanced theoretical and computational techniques. Mg^2+^ can bridge between different parts of the polymer chain or between different chains, leading to different aggregation behavior. As well, Mg^2+^ binds more strongly to the sulfonate groups than Na^+^, leading to different polymer conformations and solution properties. The divalent nature of Mg^2+^ leads to stronger electrostatic interactions, enhanced charge screening, stronger ion correlation effects, and a greater propensity for phase separation. Mg^2+^ ions are more effective at counterion condensation than Na^+^ ions due to their higher charge. This leads to a larger fraction of condensed counterions around the PVS chain. In fact, the condensation can even lead to specific binding of Mg^2+^ to the sulfonate groups. MgCl_2_ salt screens the electrostatic repulsions more effectively than NaCl at the same molar concentration. This is because the divalent Mg^2+^ ion is more effective at neutralizing the negative charges on the PVS chain.

**FIGURE 9:**
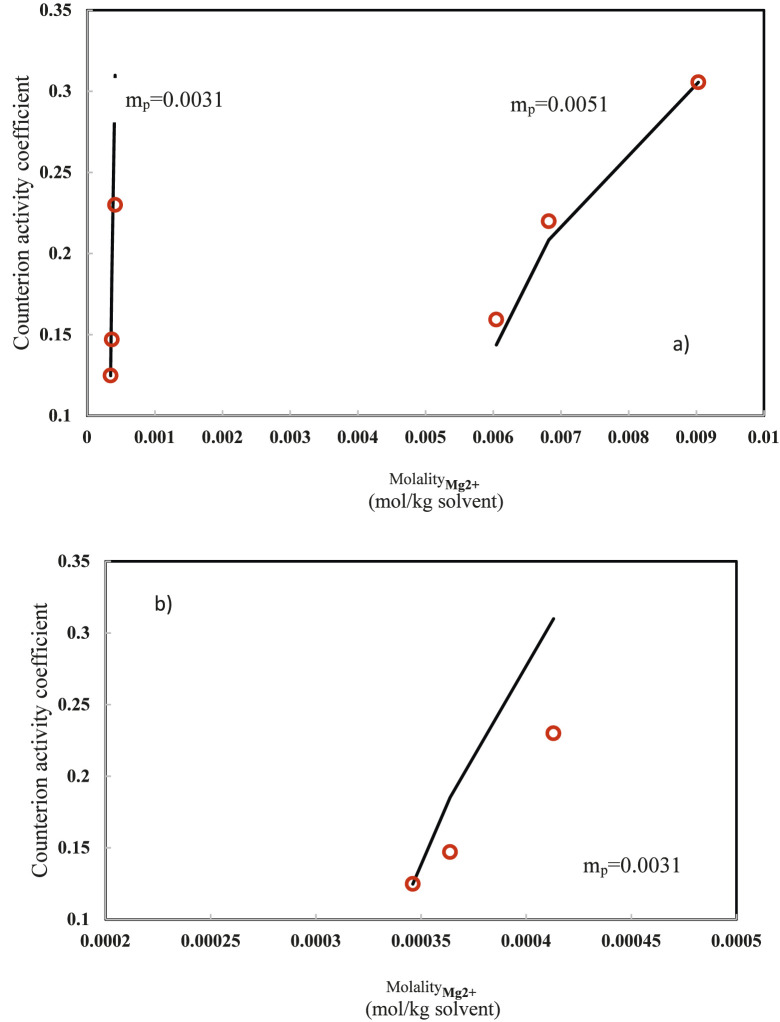
The PE-Extended UNIQUAC results (lines) and experimental data (symbols) (Lyons and Kotin, 1965) of counterion activity coefficient for MgDNA-MgCl_2_-H_2_O ternary solutions. **(b)** is zoom-in **(a)**.

In Figure 9, counterion activity coefficient in MgDNA-MgCl_2_-H_2_O system has been estimated and compared to experimental data.

DNA is a polyanion, and counterions accumulate around it to screen its negative charge. The activity of these counterions near the DNA is different from their bulk activity due to electrostatic interactions. The activity coefficients of Mg^2+^ and Cl^−^ directly affect the osmotic pressure and other thermodynamic properties of the solution. Counterion activity plays a critical role in DNA stability, conformation, and interactions with other molecules. The higher the charge density of the DNA, the greater the accumulation of counterions and the lower their activity coefficients. On the other hand, increasing the ionic strength of the solution screens the electrostatic interactions between the DNA and the counterions, leading to higher activity coefficients. Smaller ions can get closer to the DNA and screen its charge more effectively. If Mg^2+^ binds specifically to certain sites on the DNA, this will affect the distribution of ions and their activity coefficients. As shown in Figure 9, the PE-Extended UNIQUAC model estimates the counterion activity coefficient of MgDNA-MgCl_2_-H_2_O ternary solutions. The Manning parameter of MgDNA-MgCl_2_-H_2_O has been obtained 3.681. The primary effect of counterion condensation (ζ > 1) is the reduction of the DNA’s effective charge. By binding to the DNA, Mg^2+^ ions partially neutralize the negative charges on the phosphate groups. This reduces the electrostatic repulsion between different segments of the DNA molecule and between different DNA molecules in solution. The effective charge of DNA is significantly reduced, which dramatically changes its behavior in solution. By reducing the overall charge, Mg^2+^ counterion condensation reduces the electrostatic repulsion between the negatively charged phosphate groups within the DNA molecule. This helps to stabilize the DNA structure, preventing it from denaturing or unfolding. At sufficiently high concentrations, Mg^2+^ ions can act as bridges between different DNA molecules, leading to their aggregation and eventual precipitation from the solution. This occurs because a single Mg^2+^ ion can bind to phosphate groups on two different DNA molecules, effectively cross-linking them. On the other hand, the extent of counterion condensation is directly related to the concentration of Mg^2+^ in the solution. Higher concentrations lead to more condensation and more pronounced effects. The presence of other ions (such as Cl^−^) in the solution affects the activity of Mg^2+^ and the extent of counterion condensation. Higher ionic strength can screen the electrostatic interactions and reduce the amount of Mg^2+^ condensation. In Figures 9a,b, the counterion activity coefficient of MgDNA-MgCl_2_-H_2_O at two polyelectrolyte concentrations (0.0031 and 0.0051 mol/kg solvent) has been calculated. The counterion activity coefficient tends to increase by increasing the counterion concentration. The error value of model is high at m_p_ = 0.0031. The deviation observed at m_p_ = 0.0031 may be related to the presence of divalent Mg^2+^ ions, which interact more strongly with the polyion than monovalent counterions. At low polyelectrolyte concentrations, the activity coefficient becomes highly sensitive to these interactions. Since the present model represents electrostatic effects through Manning’s theory and the Debye–Hückel approach, some of the specific ion–polymer interactions may not be fully captured, leading to the observed discrepancy.

**FIGURE 10:**
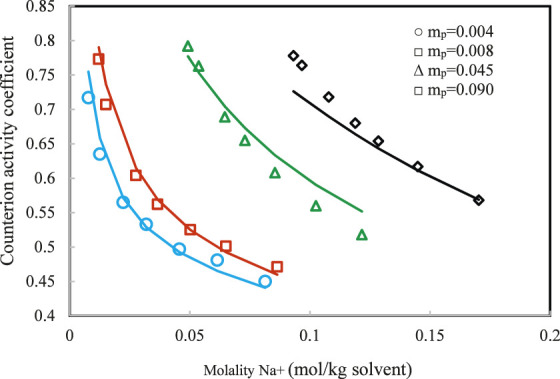
The PE-Extended UNIQUAC results (lines) and experimental data (symbols) (Podlas and Ander, 1970) of counterion activity coefficient for SA-NaCl-H_2_O ternary solutions.

A sodium alginate-NaCl-H_2_O polyelectrolyte solution is a complex system with interesting properties arising from the interplay of the charged alginate polymer and the dissolved salt. Sodium Alginate (SA) is a linear polysaccharide composed of β-D-mannuronic acid (M) and α-L-guluronic acid (G) residues. The presence of these carboxyl groups (-COO^-^) gives it a strong negative charge at physiological pH. The ratio of M and G units affects the alginate’s properties, including its ability to form gels and its interactions with other molecules. Podlasl and Ander studied the interaction of sodium and potassium ions with sodium and potassium alginate by measuring the counterion activity coefficients in aqueous solutions of the polyelectrolytes with and without added simple salt (Podlas and Ander, 1970). They showed that, the counterion activity coefficients decreased with increasing polyelectrolyte concentration at constant salt concentration, and decreased with decreasing salt concentration at a constant polyelectrolyte concentration. Yu et al. utilized the electrolyte NRTL model to estimate the counterion activity coefficient in sodium alginate-NaCl-H_2_O polyelectrolyte solution (Yu et al., 2019). They adjusted the Manning parameter and binary interaction parameters of e-NRTL using counterion activity coefficient data. Their adjusted Manning parameter was obtained 1.092. In this work, the Manning parameter has been obtained 1.123. In Figure 10, the PE-Extended UNIQUAC model results have been compared to experimental data. In this regard the counterion activity coefficient in sodium alginate-NaCl-H_2_O at four polyelectrolyte solutions have been investigated.

The average ARD% value of e-NRTL model for the counterion activity coefficient of SA-NaCl-H_2_O was obtained 1.7%. The corresponding ARD% value using the PE-Extended UNIQUAC is obtained 2.7%. As shown in Figure 10, the PE-Extended UNIQUAC results are in good agreement with experimental data.

**FIGURE 11:**
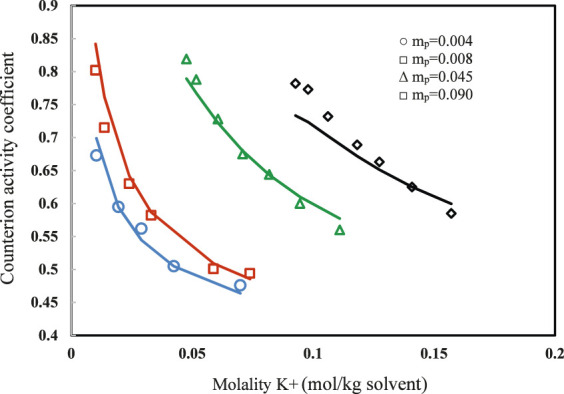
The PE-Extended UNIQUAC results (lines) and experimental data (symbols) (Podlas and Ander, 1970) of counterion activity coefficient for PA-KCl-H_2_O ternary solutions.

In Figure 11, the counterion activity coefficient of PA-KCl-H_2_O system has been estimated and compared to experimental data.

The potassium alginate-KCl polyelectrolyte system is a complex system exhibiting interesting behavior due to the interplay of the charged alginate polymer and the potassium chloride salt. The addition of KCl can lead to salting-out or salting-in effects on the alginate. Salting-out refers to the decrease in solubility of the polymer due to ion-ion interactions. Salting-in, on the other hand, leads to increased solubility due to ion-polymer interactions. The specific effect depends on the concentration of KCl, the ionic strength of the solution, and the alginate’s structure. Under specific conditions, potassium ions might interact more strongly with specific regions of the alginate, leading to the formation of complexes, which could affect the overall structure and properties of the system. K^+^ and Na^+^ differ in size and hydration properties.

A counterion with higher hydration will potentially experience a smaller effective concentration near the alginate chain. The concentration of the added salt (KCl or NaCl) will influence the ionic strength of the solution. Higher ionic strength often leads to screening effects, which can decrease the electrostatic interactions between the alginate and the counterions. This will likely affect the activity coefficients differently for K^+^ and Na^+^ depending on the specific alginate-counterion interactions. Similar to SA-KCl-H_2_O system, Yu et al. used the e-NRTL model to correlate the counterion activity coefficient of PA-KCl-H_2_O systems. The adjusted Manning parameter in e-NRTL model was about 1.092. The Manning parameter of aforementioned system in PE-Extended UNIQUAC model has been obtained 1.071. The average ARD% values of e-NRTL and PE-Extended UNIQUAC are 2.97% and 6.41% respectively.

As shown in Figures 6–11, the proposed model satisfactorily calculates the counterion activity coefficients in polyelectrolyte solutions containing added salt. The PE-Extended UNIQUAC model addresses the limitations of Manning’s limiting law and provides an improved framework for modeling such systems. It effectively correlates the activity coefficients of mobile ions in the presence of background salt. Notably, estimating the activity coefficient of a mobile ion in an electrolyte solution with added salt is a complex task, and a universal correlation is not feasible without detailed specification of the system. The activity coefficient reflects deviations from ideal behavior, indicating that ions do not behave independently due to electrostatic interactions. Several factors contribute to these deviations, with the nature and concentration of added salt playing a critical role. Among these, the most significant factor is the ionic strength of the solution. In this study, counterion condensation in polyelectrolyte solutions was investigated in the presence of MgCl_2_, KCl, and NaCl across a wide range of ionic strengths. Higher ionic strength generally leads to lower activity coefficients for all ions in the solution, primarily due to enhanced electrostatic screening. As shown in Figures 6–11, increasing ionic strength consistently resulted in a decrease in the counterion activity coefficients of the studied systems. The nature of the ions themselves has a significant impact on activity coefficients. The presence of added salt introduces new ions that interact with the original species in complex ways, which are not always adequately captured by simple electrostatic models. These interactions can be either attractive or repulsive, resulting in intricate solution behavior. Factors such as ion size, charge density, and polarizability all influence these interactions. To capture these effects, both short-range and long-range interactions between particles were modeled using the extended UNIQUAC framework. Furthermore, at higher concentrations, ion pairing (the association of oppositely charged ions into neutral or less charged complexes) can become significant, reducing the activity of free ions. The extent of ion pairing depends on the specific ion pairs and temperature. In this study, these interactions were incorporated into the development of the new thermodynamic model for aqueous polyelectrolyte solutions.

In the following section, the effects of short-range interactions, long-range interactions, and Manning theory on the counterion activity coefficient are investigated.

### 3.4 Effect of short-range, long-range, and Manning theory on activity coefficient calculation

A successful model for polyelectrolyte solutions needs to consider all these factors. Often, a combination of theoretical approaches and experimental data is needed to obtain accurate predictions of activity coefficients. The specific choice of model will depend on the specific polyelectrolyte system and the level of accuracy required. Nature of short-range Interactions arise from the specific chemical nature of the monomers in the polyelectrolyte chain, the counterions, and the solvent. Short-range interactions generally increase the activity coefficients of the ions (both the polyions and the counterions), making the solution behave more ideally. This is because these interactions effectively shield the electrostatic interactions between the charged species. Nature of long-range Interactions are primarily Coulombic (electrostatic) forces between charged species (polyions and counterions). They are long-range because they decay relatively slowly with distance. Long-range electrostatic interactions generally decrease the activity coefficients of the ions, making the solution behave less ideally. The strong attractive forces between polyions and counterions make them tend to associate, reducing their effective concentration in the solution. As described in the previous sections, nature of Manning theory is a simplified model specifically for highly charged polyelectrolytes. It focuses on the phenomenon of counterion condensation. If the charge density of the polyelectrolyte exceeds a certain threshold (defined by the Bjerrum length and the distance between charges on the polymer), counterions will condense onto the polymer chain, effectively reducing its charge. This condensation minimizes the electrostatic free energy of the system. Manning theory predicts a significant decrease in the activity coefficients of the counterions due to their condensation onto the polyelectrolyte. The activity coefficient of the polyelectrolyte itself is also affected. After condensation, the effective charge on the polyelectrolyte is reduced. While Manning Theory provides a valuable, simplified picture of counterion condensation, it relies on several assumptions that restrict its applicability and accuracy. It treats ions as point charges and neglects short-range interactions, the size of the ions, and the chemical nature of the polyelectrolyte, assumes a uniform charge distribution along the polymer chain, which is not always the case, and it predicts the activity coefficients at high charge densities, but it does not provide a complete description of the system at all concentrations. It must be noted that, all three types of interactions (short-range, long-range, and counterion condensation) are important in polyelectrolyte solutions.

**FIGURE 12:**
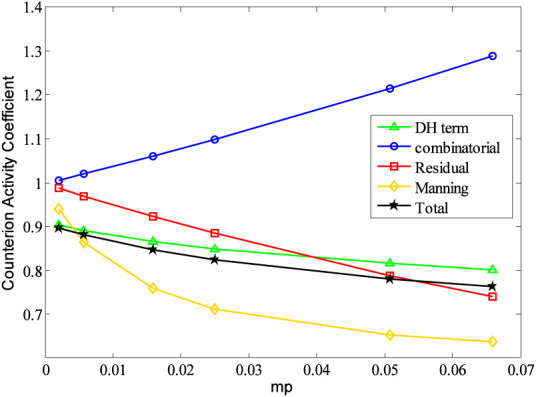
Counterion activity coefficient of PE-Extended UNIQUAC terms vs. polyelectrolyte molality of PA-KCl-H_2_O system.

In Figure 12, the activity coefficients of each contribution in the PE-Extended UNIQUAC have been depicted and compared to each other.

As shown in Figure 12, the contribution of the combinatorial term to the counterion activity coefficient increases with polyelectrolyte molality. This combinatorial term originates from the packing of molecules within the mixture, where the size and shape of molecules determine their spatial arrangement. It reflects a localized effect occurring at close proximity, describing the entropy change associated with mixing molecules of differing sizes and shapes. Importantly, this term does not directly represent specific energetic interactions. In contrast, the residual term in the UNIQUAC model explicitly accounts for energetic interactions, which can be influenced by both short- and long-range forces. Thus, while the residual term addresses the energetic contributions, the combinatorial term primarily captures the short-range effects arising from molecular size and shape differences within the mixture.

As shown in Figure 12, the residual contribution of the PE-Extended UNIQUAC model decreases with increasing polyelectrolyte concentration. Accurately describing the data at high polyelectrolyte concentrations requires accounting for short-range interactions. As the concentration increases, polymer chains move closer to each other, and short-range forces become increasingly important. Meanwhile, the increased ion concentration in the solution effectively screens long-range electrostatic interactions, reducing their influence. Consequently, at high polyelectrolyte concentrations, short-range interactions dominate the system’s behavior.

At very high concentrations, the Debye layers (the regions surrounding charged molecules where the electrostatic potential differs significantly from the bulk solution) begin to overlap. This overlap invalidates the assumptions of models based on isolated polymer chains, such as the simple Debye-Hückel theory and some extensions of Manning theory. Consequently, short-range interactions become the dominant factor governing solution behavior. These short-range forces allow counterions to bind specifically to the polymer chains and also promote polymer–polymer associations; effects that cannot be described by models based only on long-range electrostatic interactions. It is important to emphasize that accurate prediction of the osmotic coefficient, especially at high concentrations, requires incorporating short-range interactions. These short-range forces contribute significantly to the non-ideality of polyelectrolyte solutions. The results demonstrate that the phase behavior of such solutions is strongly influenced by both long-range and short-range interactions. Incorporating short-range interactions into the model improves the accuracy of phase diagram predictions. Therefore, at high polyelectrolyte concentrations, accounting for short-range interactions is essential for reliable data representation and a comprehensive understanding of the solution’s behavior. Models that consider only long-range electrostatic forces are likely to fail in capturing the complex phenomena present in these systems.

It must be noted that, the present Extended UNIQUAC–Manning framework is formulated and validated for liquid-phase equilibria in aqueous polyelectrolyte solutions, where mobile ions, counterion condensation, and long-range electrostatic interactions govern non-ideal behavior. While the extended UNIQUAC model itself has been successfully applied to solid–liquid equilibria, direct application of the current coupled approach to SLE systems would require an explicit description of the solid-phase chemical potential, including the charge state of the polyelectrolyte, possible counterion binding or immobilization, and the loss of conformational entropy upon solidification. Consequently, although extension of the proposed framework to solid–liquid equilibria is in principle possible, it would require additional model development and is beyond the scope of the present study.

## 4 Conclusion

In this work, the extended UNIQUAC model was further developed for polyelectrolyte solutions by incorporating the Manning theory to account for counterion condensation. Long-range electrostatic interactions were modeled using the Debye-Hückel theory within the extended UNIQUAC framework, while short-range interactions among all species were captured by the combinatorial and residual terms of the UNIQUAC model. Model parameters were adjusted based on experimental data for counterion activity coefficients in polyelectrolyte solutions with added salts and osmotic coefficients in solutions without added salt. The extended UNIQUAC parameters, the Manning parameter was also optimized. A total of fourteen polyelectrolyte-containing systems were investigated in this study. The average absolute relative deviation (ARD%) of the counterion activity coefficient for seven polyelectrolyte solutions with added salts was found to be 4.6%. These results demonstrate that the proposed PE-Extended UNIQUAC model can satisfactorily predict both the counterion activity coefficients and osmotic coefficients of polyelectrolyte solutions in the presence of salt. The model effectively captures the impact of polymer charge, confirming its applicability to both neutral and charged polymer systems. Additionally, the effects of the Manning contribution, as well as short-range and long-range interactions, on the counterion activity coefficient were examined. The findings highlight that accurate prediction of the osmotic coefficient, especially at high concentrations, necessitates consideration of short-range forces. In this context, the combinatorial and residual terms of the proposed model play a crucial role in the thermodynamic modeling of polyelectrolyte-containing systems.

## Data Availability

The original contributions presented in the study are included in the article/Supplementary Material, further inquiries can be directed to the corresponding author.
