# Supplementary material for: Development a new thermodynamic model for aqueous polyelectrolyte solutions: extended UNIQUAC coupled with Manning’s theory
Source: Front Chem. 2026 Jul 8;14:1853816. doi: 10.3389/fchem.2026.1853816 (PMC13389867; doi:10.3389/fchem.2026.1853816)
Supplement: Supplementary file 1 [file Supplementaryfile1.docx]

**Supplementary Appendix A. Extended UNIQUAC for poly electrolyte containing systems**

The Extended UNIQUAC model combined with the Debye-Hückel theory was employed as a reference framework to describe the thermodynamic behavior of electrolyte-containing systems. In the Extended UNIQUAC framework, the excess Gibbs energy is expressed as the sum of a short-range contribution, described by the UNIQUAC model, and a long-range electrostatic contribution, represented by the Debye-Hückel theory. The UNIQUAC term accounts for combinatorial and residual interactions between all species present in the system, while the Debye-Hückel term describes electrostatic interactions among charged species as a function of ionic strength.

For the purpose of comparison, polyelectrolytes were treated using a fully dissociated electrolyte assumption, whereby each charged repeating unit of the polymer was considered to dissociate completely into its corresponding counterions. As a result, the polyelectrolyte was represented as an assembly of independent ionic species, neglecting connectivity effects along the polymer backbone, counterion condensation phenomena, and specific ion-polymer correlations. The Debye-Hückel contribution was applied to all ionic species using effective ionic charges and a mean ionic strength calculated from the fully dissociated species. No Manning-type condensation term or chain-length-dependent electrostatic correction was included. Consequently, electrostatic interactions were modeled solely through the classical Debye-Hückel formulation, which is known to be valid primarily at low to moderate ionic strengths.

Due to these simplifications, the Extended UNIQUAC approach is not intended to provide a rigorous description of polyelectrolyte thermodynamics. Instead, it serves as a first-order approximation and a benchmark model, enabling a quantitative comparison with more advanced and physically consistent polyelectrolyte models, such as the PE-UNIQUAC model proposed in this work and the pePC-SAFT equation of state.

The r_i_, q_i_ and interaction parameter (U_ij_) between polyion and water have been considered as adjustable parameters. Similar OF (Eq. 38) has been utilized to adjust the model parameters. In Tables A1 and A2 the aforementioned parameters have been presented.

Table A1. Volume parameter (*rᵢ*) and surface area parameter (*q_i_*) of extended UNIQUAC.

| Components | *rᵢ* | *q_i_* |
| --- | --- | --- |
| H_2_O | 0.92 | 1.40 |
| ${Na}^{+}$ | 1.4034 | 1.199 |
| $K^{+}$ | 1.86 | 1.71 |
| ${Mg}^{2+}$ | 3.55 | 0.805 |
| ${NH}_{4}^{+}$ | 4.815 | 4.6028 |
| ${Cl}^{-}$ | 10.82 | 10.4 |
| ${PA5}^{-}$ | 3.691 | 16.92 |
| ${PA15}^{-}$ | 3.155 | 42.25 |
| ${PES2}^{-}$ | 4.778 | 41.34 |
| ${PES10}^{-}$ | 3.491 | 30.82 |
| ${PMA6}^{-}$ | 3.531 | 36.561 |
| ${PMA15}^{-}$ | 4.927 | 81.322 |

Table A2. Interaction parameters of Extended UNIQUAC model between polyions and water.

| Polyion | H_2_O |
| --- | --- |
| ${PA5}^{-}$ | -1307.585 |
| ${PA15}^{-}$ | -1146.551 |
| ${PES2}^{-}$ | -963.192 |
| ${PES10}^{-}$ | -1110.368 |
| ${PMA6}^{-}$ | -1143.150 |
| ${PMA15}^{-}$ | -1102.990 |
|  |  |

Similar to the PE-Extended UNIQUAC model, the osmotic coefficient and water activity of some polyelectrolyte systems have been studied. As shown in Tables A1 and A2, the polyelectrolyte must be modeled as fully dissociated electrolyte in the Extended UNIQUAC model. For each system three model parameters were adjusted using experimental data. In Table A3 the ARD% values of several systems have been reported and compared to PE-Extended UNIQUAC model.

Table A3. The average relative deviation (ARD%) of osmotic coefficient and water activity of PE-Extended UNIQUAC and Extended UNIQUAC models.

| Components | PE-Extended UNIQUAC | | | Extended UNIQUAC | |
| --- | --- | --- | --- | --- | --- |
|  | $\phi$ |  | $a_{w}$ | $\phi$ | $a_{w}$ |
| NaPA5 | 5.6 |  | 0.21 | 9.13 | 3.13 |
| NaPA15 | 5.8 |  | 0.23 | 6.81 | 4.71 |
| NaPES2 | 7..5 |  | 0.36 | 14.80 | 5.92 |
| NaPES10 | 8.2 |  | 0.19 | 10.75 | 4.11 |
| NaPMA6 | 9.2 |  | 0.43 | 7.53 | 3.31 |
| NaPMA15 | 6.9 |  | 0.18 | 8.51 | 3.10 |
| **Average Error** | **5.95** |  | **0.27** | **9.59** | **4.05** |

Table A3 compares the performance of the PE-Extended UNIQUAC model and the conventional Extended UNIQUAC framework for several sodium polyelectrolyte systems. The comparison is based on deviations in the osmotic coefficient (ϕ) and water activity (a_w_). For all investigated systems, the PE-Extended UNIQUAC model yields significantly lower deviations in both osmotic coefficients and water activity compared to the Extended UNIQUAC model. For example, in the NaPA5 and NaPA15 systems, deviations in ϕ are reduced from 9.13 and 6.81 to 5.6 and 5.8, respectively, while the corresponding errors in water activity decrease by nearly an order of magnitude. Similar trends are observed for NaPES and NaPMA systems, where the Extended UNIQUAC model consistently overpredicts deviations, particularly for higher charge densities and molecular weights.

The performance of the Extended UNIQUAC approach can be attributed to its underlying assumption of fully dissociated and independent ionic species. This simplification neglects essential polyelectrolyte-specific effects such as chain connectivity, electrostatic correlations along the polymer backbone, and counterion condensation. As a result, long-range electrostatic interactions are insufficiently captured, especially at moderate to high ionic strengths. It should also be noted that the PE-UNIQUAC model involves a larger number of adjustable parameters compared to the conventional Extended UNIQUAC framework, which may partially contribute to the observed reduction in deviations. Nevertheless, the improved performance is not solely a consequence of increased parameterization but primarily reflects the inclusion of physically relevant polyelectrolyte effects that are absent in classical electrolyte models.

In Figure A1 the osmotic coefficient of NaPA5 and NaPES2 for two Extended UNIQUAC and PE-UNIQUAC model has been estimated and compared to experimental data.

a)

b)

Figure A1. Osmotic coefficient of (a) NaPA5 and (b) NaPES2 obtained by the Extended UNIQUAC (dashed line) and PE-Extended UNIQUAC (solid line).

Figure A1 presents the osmotic coefficients of (a) NaPA5 and (b) NaPES2 as a function of mole fraction, comparing calculations from the Extended UNIQUAC model with Debye-Hückel term (dashed line) and the PE-Extended UNIQUAC model (solid line) against experimental data (symbols). For both systems, the models capture the overall increasing trend of the osmotic coefficient with concentration. The PE-Extended UNIQUAC model shows slightly improved agreement at higher concentrations, while the Extended UNIQUAC model provides a reasonable description across the full concentration range.

**Supplementary Appendix B. Electrolyte PC-SAFT EoS for polyelectrolyte containing systems**

In this work, polyelectrolyte-containing systems are modeled using the electrolyte PC-SAFT equation of state (EoS) based on a fully dissociated representation of the polyelectrolyte. The polymer is treated as a chain of charged monomeric segments, each carrying a fixed negative charge, while the corresponding counterions are modeled as independent ionic species in solution. No association between the charged polymer segments and counterions is assumed, and all ionic species are considered to be completely dissociated over the investigated concentration range. The polyelectrolyte is represented by an effective segment number and segment parameters derived from the corresponding neutral polymer, with the electrostatic contribution accounting for the additional non-ideality introduced by the presence of charges. This fully dissociated approach provides a simplified yet physically consistent description of polyelectrolyte solutions and allows direct comparison with electrolyte activity and osmotic coefficient data.

It must be noted that, in the pePC-SAFT framework proposed by Naeem and Sadowski [9], the influence of charged monomers is incorporated directly into the reference term through a charged-hard-chain contribution, while the reduction in effective polymer charge due to counterion condensation is explicitly considered. The remaining free counterions are treated via a Debye-Hückel electrostatic term, ensuring a physically consistent description of electrostatic interactions in polyelectrolyte systems. For more detail refer to [9].

In this work, a simplifying assumption is introduced to reduce the complexity of the pePC-SAFT equation of state, and a simplified EOS-based model is proposed for polyelectrolyte-containing systems. The residual Helmholtz free energy of the ePC-SAFT EoS is as follows [29]:

| $\frac{A^{res}}{Nk_{B}T}=\frac{A^{hc}}{Nk_{B}T}+\frac{A^{disp}}{Nk_{B}T}+\frac{A^{assoc}}{Nk_{B}T}+\frac{A^{elec}}{Nk_{B}T}$ | (A1) |
| --- | --- |

Here, the superscripts *res*, *hc*, *disp*, *assoc,* and *elec* represent the residual, hard-chain, dispersion, association, and electrostatic contributions, respectively.

The hard-chain ($A^{\text{hc}}$) and dispersion ($A^{\text{disp}}$) terms are given by [29]:

| $\frac{A^{hc}}{Nk_{B}T}=\bar{m}\frac{A^{hs}}{Nk_{B}T}-\sum_{i} x_{i}\left( m_{i}-1 \right)\ln g_{ii}^{hs}(\sigma_{ii})$ | (A2) |
| --- | --- |
| $\frac{A^{hs}}{Nk_{B}T}=\frac{1}{\xi_{0}}\left[ \frac{3\xi_{1}\xi_{2}}{(1-\xi_{3})}+\frac{\xi_{2}^{3}}{\xi_{3}{(1-\xi_{3})}^{2}}+\left( \frac{\xi_{2}^{3}}{\xi_{3}^{2}}-\xi_{0} \right)\ln(1-\xi_{3}) \right]$ | (A3) |
| $\frac{A^{disp}}{Nk_{B}T}=-2\pi\rho I_{1}\left( \eta,\bar{m} \right)\bar{m^{2}\epsilon\sigma^{3}}-\pi\rho\bar{m}C_{1}I_{2}(\eta,\bar{m})\bar{m^{2}\epsilon^{2}\sigma^{3}}$ | (A4) |

The association term describes interactions that are highly directional, such as hydrogen bonds and other specific intermolecular forces. This term plays a key role in accurately representing fluids that exhibit strong associating behavior. The original theoretical approach for this contribution was introduced by Chapman et al. [30]:

| $\frac{A^{assoc}}{Nk_{B}T}=\sum_{i} x_{i}\left[ \sum_{A_{i}} \left[ \ln X^{A_{i}}-\frac{X^{A_{i}}}{2} \right]+\frac{M_{i}}{2} \right]$ | (A5) |
| --- | --- |

In this framework, $x_{i}$ represents the mole fraction of component $i$ within the mixture, $M$ denotes the number of association sites per molecule, and $X_{A}^{i}$ corresponds to the fraction of component $i$ molecules whose site $A$ remains unbonded. The values of $X_{A}^{i}$ is given by:

| $X^{A_{i}}=\frac{1}{1+\rho\sum_{i} x_{i}\sum_{B_{j}} X^{B_{i}}\Delta^{A_{i}B_{j}}}$ | (A6) |
| --- | --- |

where $\Delta^{A_{i}B_{j}}$ (interaction strength) between association sites $A$ and $B$ is defined as follows:

| $\Delta^{A_{i}B_{j}}={}_{ij}^{hs}(d_{ij}^{+})\left[ \exp\left( \frac{\varepsilon^{A_{i}B_{j}}}{k_{B}T} \right)-1 \right]\sigma_{ij}^{3}\kappa^{A_{i}B_{j}}$ | (A7) |
| --- | --- |

In this context, $\varepsilon^{A_{i}B_{j}}$ and $\kappa^{A_{i}B_{j}}$ refer to the energy of association and interaction volume.

The Debye–Hückel term (electrostatic) was defined as:

| $\frac{A^{elec}}{Nk_{B}T}=-\frac{k}{12\pi k_{B}TD}\sum_{i} x_{i}q_{i}^{2}\chi_{i}$ | (A8) |
| --- | --- |

In this equation D refers to dielectric constant of solution. Further details on the parameters are provided in Reference [31].

In the present work, the polyion parameters, such as segment number, segment diameter, and segment energy were considered adjustable for modeling purposes. The following objective function has been utilized to adjust the model parameters:

| $OF=w_{1}\sum_{i} \left[ \frac{\gamma_{i}^{+,exp}-\gamma_{i}^{+,calc}}{\gamma_{i}^{+,exp}} \right]^{2}+w_{2}\sum_{i} \left[ \frac{\phi^{exp}-\phi^{calc}}{\phi^{exp}} \right]^{2}$ | (A9) |
| --- | --- |

where $w_{1}$ and $w_{2}$ refer to weight parameters of OF; refer to Table 2.

In Table A4 the electrolyte PC-SAFT parameters have been reported. It must be noted that the water and other specific ion parameters (for Na^+^, Cl^-^, and….) are reported in the reference [9, 31]. The water has been modeled as an association component with two association sites; please refer to references [9, 31].

Table A4. The polyion parameters of the ePC-SAFT EoS.

| Polyion | m/Mw(mol/g) | $\sigma$(A) | $\varepsilon/{k_{B}}$(K) |
| --- | --- | --- | --- |
| ${PA}^{-}$ | 0.01287 | 3.8120 | 1313.22 |
| ${PES}^{-}$ | 0.00766 | 3.8496 | 1372.00 |
| ${PMA}^{-}$ | 0.00146 | 3.7519 | 1227.39 |

Table A5 compares the performance of the PE-Extended UNIQUAC model and the ePC-SAFT EoS for estimating the osmotic coefficient ($\phi$) and water activity ($a_{w}$) of various polyelectrolyte systems. The results indicate noticeable differences in accuracy between the two models across all investigated components.

Table A5. The average relative deviation (ARD%) of osmotic coefficient and water activity of PE-Extended UNIQUAC.

| Components | PE-Extended UNIQUAC | | | ePC-SAFT EoS | |
| --- | --- | --- | --- | --- | --- |
|  | $\phi$ |  | $a_{w}$ | $\phi$ | $a_{w}$ |
| NaPA5 | 5.6 |  | 0.21 | 14.3 | 4.23 |
| NaPA15 | 5.8 |  | 0.23 | 9.56 | 5.12 |
| NaPES2 | 7..5 |  | 0.36 | 16.3 | 6.01 |
| NaPES10 | 8.2 |  | 0.19 | 4.53 | 2.11 |
| NaPMA6 | 9.2 |  | 0.43 | 15.40 | 5.22 |
| NaPMA15 | 6.9 |  | 0.18 | 17.61 | 5.83 |
| **Average Error** | **5.95** |  | **0.27** | **12.95** | **4.75** |

Overall, the PE-Extended UNIQUAC model demonstrates superior agreement with the experimental data, yielding significantly lower average deviations for both properties. The average error in the osmotic coefficient calculated by PE-Extended UNIQUAC is 5.95, compared to 12.95 obtained using ePC-SAFT. A similar trend is observed for water activity, where the average error is reduced from 4.75 with ePC-SAFT to 0.27 using PE-Extended UNIQUAC.

The results confirm that PE-Extended UNIQUAC provides a more reliable description of both osmotic and activity properties for the studied systems, making it a preferable modeling approach for polyelectrolyte-water mixtures under the conditions examined in this work. In Figure A2 the osmotic coefficient of NaPA5 and NaPA15 has been calculated and compared o experimental data.

a)

b)

Figure A2. Osmotic coefficient of (a) NaPA5 and (b) NaPA15 obtained by the ePC-SAFT EoS (dashed line) and PE-Extended UNIQUAC (solid line).

The results demonstrate that both the Extended UNIQUAC and electrolyte PC-SAFT models are capable of describing polyelectrolyte-containing systems with acceptable accuracy. However, among the models considered, the PE-Extended UNIQUAC model provides the best overall performance. Furthermore, the proposed electrolyte PC-SAFT framework shows potential for extension to additional polyelectrolyte-containing systems in future studies. This is because the thermodynamic properties of polyelectrolyte-containing systems can be predicted directly using equation of state-based models.
